# Photoelectric Imaging of the Optically Inactive Charge State of Silicon‐Vacancy Defects in Diamond

**DOI:** 10.1002/advs.76933

**Published:** 2026-08-06

**Authors:** Ilia Chuprina, Gergő Thiering, Emilie Bourgeois, Anna Ermakova, Dmitry Budker, Adam Gali, Milos Nesladek, Petr Siyushev, Fedor Jelezko

**Affiliations:** ^1^ Institute for Quantum Optics Ulm University Ulm Germany; ^2^ HUN‐REN Wigner Research Centre for Physics Budapest Hungary; ^3^ IMOMEC division IMEC Diepenbeek Belgium; ^4^ Institute for Materials Research (IMO) Hasselt University Diepenbeek Belgium; ^5^ Royal Belgian Institute for Space Aeronomy (BIRA‐IASB) Brussels Belgium; ^6^ Helmholtz‐Institut Mainz Mainz Germany; ^7^ Johannes Gutenberg‐Universität Mainz Mainz Germany; ^8^ GSI Helmholtzzentrum für Schwerionenforschung GmbH Darmstadt Germany; ^9^ Department of Physics University of California Berkeley USA; ^10^ Department of Atomic Physics Institute of Physics Budapest University of Technology and Economics Budapest Hungary; ^11^ MTA–WFK Lendület “Momentum” Semiconductor Nanostructures Research Group Budapest Hungary; ^12^ Department of Biomedical Technology Faculty of Biomedical Engineering Czech Technical University in Prague Kladno Czech Republic; ^13^ Center for Integrated Quantum Science and Technology (IQST) Ulm Germany

**Keywords:** crystallographic defect, diamond, electron paramagnetic resonance, materials science, optoelectronics, photoelectric effect, quantum sensor, semiconductor, vacancy defect

## Abstract

The investigation of optically active point defects in wide‐bandgap semiconductors is driven by the development of solid‐state quantum technologies. Such atom‐like defects are used for the realization of quantum registers and quantum sensors. Reproducible engineering of even well‐established defects with predictable properties remains challenging, as the local environment and complex charge dynamics limit their performance and applicability. Furthermore, not all defects are optically accessible, which hinders our ability to study them. Here, we use the photoelectric detection technique to study switching and control of desired double and single charge states of silicon vacancy (SiV) defect in diamond. We demonstrate the first direct imaging of the dark charge state of SiV, which cannot be addressed by optical methods or electron paramagnetic resonance. We discuss a model for SiV charge state conversion involving proximal substitutional nitrogen defects. These findings are important for the charge state control of the entire family of group‐IV defects in diamond.

## Graphical abstract

Although the doubly negative charge state of the SiV center in diamond is predicted to ionize only in the ultraviolet and is therefore considered optically inaccessible, we demonstrate visible‐range photoelectric imaging enabled by a previously unrecognized regime of laser‐assisted tunneling.

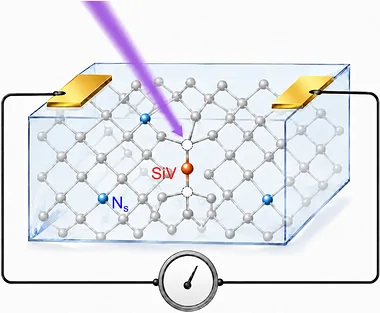

## 1 Introduction

Intensive investigation of group‐IV defects in diamond is strongly linked to their remarkable optical properties, which make them attractive candidates for quantum information processing. Particularly, they emit ≈70% of light associated with the zero‐phonon line  [1, 2] and, due to $D_{3d}$ symmetry, they are robust against spectral jumps and diffusion  [3, 4]. Among these defects, the silicon‐vacancy (SiV) centers are the most studied ones because their production is well established. Their fabrication can be achieved not only by ion implantation but also by the incorporation of Si atoms into the diamond crystal during growth. In‐grown defects usually exhibit the best properties compared to implanted ones, and thus they can be considered almost ideal case for studies. At the same time, multiple problems remain associated with the reliable fabrication of group‐IV defects, such as low activation yield  [5], or blinking  [6] of luminescence. Understanding these problems is strongly related to charge dynamics. Photoelectric detection, which has been recently developed for nitrogen‐vacancy centers in diamond  [7] might be a powerful tool to investigate and visualize other defects. The method of photoelectric readout of the electron spin state was also extended to defects in silicon‐carbide  [8] and readout of nuclear spins in diamond  [9, 10]. The main requirement for the application of this technique is that the defect should be prone to photoionization or undergo other charge dynamics that result in the generation of free carriers.

In this work, we report direct observation of non‐fluorescent point defects in diamond using a photoelectric detection technique. Using a combination of optical and photoelectric methods, we find that the “dark” defects are related to the SiV defect. The appearance of both luminescent SiV and non‐luminescent centers is strongly influenced by the applied bias voltage, which allows for their activation and deactivation. We attribute these optically inactive centers to the doubly negative charge state of silicon‐vacancy (${\mathrm{SiV}}^{2-}$) and discuss a model supported by first‐principles calculations that can explain the observed behavior. Our work provides further insight into the nature of the charge state dynamics of SiV centers and can be extrapolated to the whole class of group‐IV defects.

## 2 Results

### 2.1 Charge‐State Switching

For the experiment, we used a 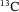 isotopically depleted diamond layer grown on top of a [111]‐oriented IIa substrate by the chemical vapor deposition technique. Incorporation of SiV centers was achieved during the growth. The concentration of nitrogen is not precisely known, but comparing the number of nitrogen‐vacancy centers in this sample to the typical electronic grade diamonds from “Element Six”, it can be estimated to be at the level of ∼10 ppb. Titanium electrodes with inter‐electrode spacing of 50 μm were produced on the diamond surface by photolithography method. The detailed fabrication procedure can be found in Ref.  [7]

The SiV center consists of an interstitial Si atom located between two neighboring vacant carbon sites, resulting in a split‐vacancy configuration. In intrinsic diamonds, the center is usually found in its negative charge state (${\mathrm{SiV}}^{-}$)  [11], however, other charge states are possible  [12, 13]. Boron doping allows one to obtain a neutral charge state (${\mathrm{SiV}}^{0}$)  [14], which was detected down to a single impurity level  [15]. In general, the charge state of the defect is governed by the position of the Fermi level with respect to the charge transition levels of a defect  [16]. Applying a potential to the electrodes, diamond bands and the charge transition levels of the defect undergo bending  [17]. This can result in changing the charge state of the defect near the electrodes. Figure 1a shows three confocal scans of the same area near one electrode (see Supporting Information for sample geometry). Without any voltage, few spots are visible corresponding to ${\mathrm{SiV}}^{-}$, which was confirmed by spectra (Figure 1b). When a positive potential is applied to the electrode, the number of ${\mathrm{SiV}}^{-}$ centers decreases. Also, luminescence significantly reduces from some spots that do not turn off completely. In contrast, application of negative voltage leads to the activation of numerous ${\mathrm{SiV}}^{-}$ defects. The band‐bending picture suggests that most of the SiV centers in diamonds occupy a charge state different from that of the negative (see Supporting Information). This fact agrees well with a previous first principles study showing the existence of doubly negative charge state for SiV defect  [18] where the accurate charge transition level of ${\mathrm{SiV}}^{2-}$ and ${\mathrm{SiV}}^{-}$ lies at approximately 2.45 eV above the valence band maximum (see Ref.  [19] for the method and Figure S12 for the result). However, charge switching was observed more than 10 μm away from the electrode, which is too far to be explained by band bending within the depletion region  [20]. It was also shown that for low concentration of impurities, the Fermi level does not unambiguously define the defect's charge state  [21]. Therefore, defects can co‐exist in different charge states in the same crystal where illumination with light or free charges may initiate charge dynamics and exchange of charge carriers that are otherwise blocked in thermal equilibrium. As explained below, the band bending does not play an important role in this case.

**FIGURE 1:**
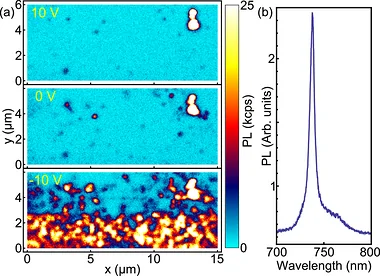
Voltage depended luminescence from SiV centers. (a) Three confocal scans of the same area near the electrode. The luminescence of the ${\mathrm{SiV}}^{-}$ centers increases when potential of the closest electrodes varies from 10 V (upper panel) to 0 V (middle panel) and ‐10 V (lower panel). (b) Typical ${\mathrm{SiV}}^{-}$ spectrum measured for multiple luminescence spots obtained for different voltages.

### 2.2 Photoelectric Defect Imaging

Setting the electric potential to 4 V at one electrode, while another is set to the ground, we scanned the area between these electrodes. Simultaneously obtained photoluminescence (PL) and photoelectric maps revealed two types of defects: those visible in both optical and photoelectric scans (referred to as *first type*) and those visible only in the photoelectric image (referred to as *second type*). An example of the scans is shown in Figure 2a,b. The overall number of defects of the *first type* was less than 1% with respect to the *second type* and no correlation between depth of the centers or proximity to the electrode influence defect's type was observed. To verify that the optically “invisible” centers do not emit at shorter wavelengths, we tuned an optical parametric oscillator (OPO) to 450 nm and opened the detection window from 500 nm. In this configuration “dark” centers remained invisible in the optical scan, while the photoelectric image was preserved.

**FIGURE 2:**
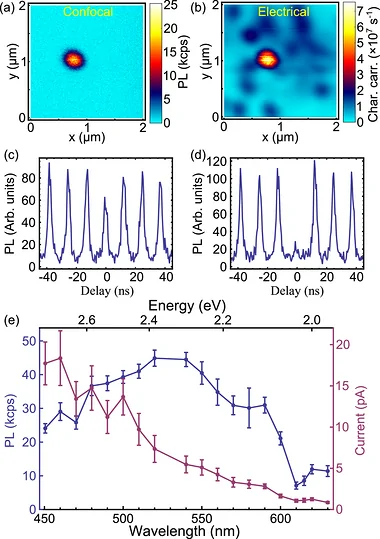
Imaging of optically active and inactive centers. (a) Confocal scan of an area between electrodes. The OPO excitation power is 8 mW at wavelength of 455 nm. The applied electric potential is 4 V. (b) Corresponding photoelectric image of the same area recorded under the same conditions. (c) and (d) Second‐order autocorrelation function obtained for the potential of 0  and 5 V, respectively. (e) PLE (blue) and photoelectric (purple) spectra of the SiV center (*first type*) depicted in (a,b). The excitation power is 8 mW over entire excitation range and the voltage is 4 V.

First, we characterized a spot visible in both images (*first type*). The PL emission from this spot exhibited typical ${\mathrm{SiV}}^{-}$ spectrum. We note that the second‐order autocorrelation function at zero delay time was greater than 0.5 indicating that there are at least two centers in the same spot. However, it is possible with further increase of the potential to suppress PL from some of the centers within the spot so that the autocorrelation function falls below 0.5 (Figure 2c,d). Therefore, we deliberately use the word “spot” rather than “color center” because we cannot unambiguously ensure that the PL or photoelectric signal originates from a single defect. In order to find an optimal excitation wavelength for the observation of the photoelectric signal, the OPO was tuned in the range of 450–650 nm with the increment of 10 nm. The excitation power of 8 mW was well below saturation  [3]. The collected data points corresponding to the electrical and optical signals constitute photoelectric and photoluminescence excitation (PLE) spectra (Figure 2e). For the wavelengths above 550 nm, both PLE and photoelectric signals increase with increasing photon energy. However, for shorter wavelengths optical absorption decreases, while photocurrent continues to grow monotonically. Also, this SiV was well excited at 705 nm, but the photoelectric signal was absent.

### 2.3 Assignment of Optically Inactive Defects to the SiV

The *second type* of defects are not visible in the optical image when positive potential is applied (Figure 3a,b). However, the situation changes when the voltage is reversed: the ${\mathrm{SiV}}^{-}$ centers start to luminesce (Figure 3c) whereas the photoelectric signal measured at the position of these centers is strongly suppressed (Figure 3d). The amplitude of the photocurrent drops by more than an order of magnitude, so that for most of the spots the signal becomes buried in noise. Figure 3e shows the dependence of the photoelectric and photoluminescence signals on the applied voltage measured at one chosen spot. We note that the point at which the signals switch can vary in both directions around 0 V depending on the particular spot and also the prehistory of the applied voltage. If the former is likely related to the defect's environment, the latter is associated with the imperfection of contacts (charging of nearby deep‐level traps) resulting in the photocurrent hysteresis. Similar to the *first type* of centers, the photocurrent increases for the excitation at the shorter wavelengths. However, at longer wavelengths the photocurrent excitation profile exhibits a threshold at around 540 nm (Figure 3). Comparing the electrical image in Figure 3b at 5 V and the optical image in Figure 3c at ‐5 V, high co‐localization of the spots can be observed. This suggests that the “dark” defects are related to SiV. Additional evidence supporting this assumption is the following. Varying voltage to the point where the PL emission tends to disappear, many centers start to blink. At the switching point, there is a small overlap region, when optical and photoelectric signals can be observed simultaneously (Figure 3e). At this voltage, jumps in the photoelectric and optical signals can be observed. Figure 3g displays an example of such synchronously recorded time traces, exhibiting strong correlation (see Supporting Information). This also suggests that the photocurrent and PL emission originate from the same defect, although, in a different charge state.

**FIGURE 3:**
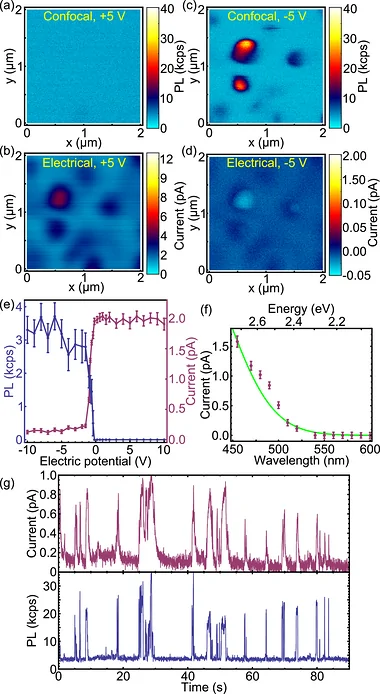
Switching between charge states. (a) and (b) Confocal and corresponding photoelectric scans with excitation wavelength 455 nm, 10 mW power, and 5 V potential. (c) and (d) Confocal and photoelectric images of the same area and the same excitation parameters, but potential is switched to ‐5 V. (e) Switching of photoelectric and optical signals measured at the same spot upon reversing applied voltage. The data points are amplitudes of the signals on top of background fitted with Gaussian function. (f) Wavelength dependent photocurrent obtained for a “dark” defect. The green curve depicts the theoretically predicted charge transfer probability by means of *ab‐initio* density functional calculations of the ${\mathrm{SiV}}^{2-}$+$N_{S}^{+}$ ⟶ ${\mathrm{SiV}}^{-}$+$N_{S}^{0}$ charge transfer (see Supporting Information for details). (g) An example of blinking photoelectric and optical time traces recorded synchronously from the same spot at near‐zero voltage.

## 3 Discussion

### 3.1 Discussion of Possible Charge‐Conversion Mechanisms

As follows from the above, the defects of the *first* and *second types* are related to the SiV centers. However, the photocurrent origin should still be discussed. Generation of charge carriers can occur in two alternative pathways. The first can involve photoionization of the ${\mathrm{SiV}}^{-}$ so that it switches to ${\mathrm{SiV}}^{0}$ and then restores the negative state by acquiring an electron from elsewhere. The second can occur via transition of an electron from the valence band to the excited state of a ${\mathrm{SiV}}^{-}$, converting it to ${\mathrm{SiV}}^{2-}$ and then the defect captures a hole from elsewhere or experiences photoionization so that the single negative charge state is restored. As discussed earlier, the transition level between double and single negative charge states of the SiV center lies below the middle of the bandgap and therefore below the Fermi level. Thus, most of the centers in thermodynamic equilibrium should be in ${\mathrm{SiV}}^{2-}$ and only a small amount of them is found in the ${\mathrm{SiV}}^{-}$. Near the positively biased contact, the diamond bands bend upward, such that the Fermi level remains above the (2−|−) transition level. Additional evidence against activation of the neutral charge state is the absence of the characteristic zero‐phonon line near 946 nm in the PL spectrum (see Supporting Information). Moreover, the photon‐excitation energy ranging from 1.76 to 2.76 eV (705–450 nm) used in these experiments is not enough to promote an electron from the excited state of ${\mathrm{SiV}}^{-}$ to the conduction band in a single‐photon process  [22]. This would require approximately 3.9 eV (318 nm).

The remaining possibility is therefore a charge cycle involving the doubly negative charge state. However, direct photoionization of ${\mathrm{SiV}}^{2-}$ also cannot explain the experiment. The (2−|−) transition level lies 2.45 eV above the valence‐band maximum; therefore, direct photoionization of ${\mathrm{SiV}}^{2-}$ to ${\mathrm{SiV}}^{-}$ requires a minimum photon energy of 2.95 eV (420 nm). This value is inconsistent with the experimentally observed threshold of approximately 2.3 eV (540 nm). The proposed model in Ref.  [23] that aims to explain the luminescence recovery of tin‐vacancy (SnV) center after bleaching, cannot be applied either. This model relies on the auxiliary defect (divacancy), which acquires an electron from the valence band upon laser excitation, while the generated hole is captured by the SnV center. This process implies the hole‐exchange between these defects. Recently, another similar model was proposed for ensemble of SiV centers, which relies on hole generation by exciting electrons from the valence band to the Fermi level of the positive electrode or the same hole ionization from divacancy  [24]. Such a hole‐exchange mechanism, however, is inconsistent with our photocurrent‐imaging results. We observe that charge carriers are generated locally at the defect position under optical illumination, leading to an increase in photocurrent. The hole‐exchange process would not lead to a change in photocurrent because the number of trapped and released charge carriers remains constant. Therefore, a defect that appears in photocurrent as a point‐like source must generate a free electron–hole pair during its charge‐conversion cycle. It is also important that in our work the sample was not implanted and should not contain divacancy defects. These considerations motivate an alternative mechanism.

We propose that only those SiV defects that have nearby auxiliary defects exhibit the observed photocurrent. These auxiliary defects should be just a few lattice constants away from SiV but, still far enough that the energy levels of different defects are unaffected, i.e., no new defect is formed instead of SiV. Furthermore, the auxiliary defect should have an electronic level close enough to the conduction band to be photoionized by photons with energies of at least 2.3 eV. Substitutional nitrogen defects, $N_{s}$, are natural candidates, because their optical ionization threshold is 2.2 eV  [25, 26, 27], above the threshold observed in our experiments. In this picture, an electron from $e_{g}$ orbital of ${\mathrm{SiV}}^{2-}$ can hop to a nearby $N_{s}^{+}$. A related qualitative model was developed for NV – $N_{s}$ pair  [28, 29], suggesting that the electron from $N_{s}$ hops to the proximal NV defect giving it a negative charge state. The same works also argued that the reverse process, in which an electron hops from ${\mathrm{NV}}^{-}$ to the $N_{s}^{+}$, is possible. However, the hopping rate between neighboring defects should be influenced by the energy mismatch of their levels. The SiV (2−|−) charge transition level is 2.45 eV above the valence band maximum, while the (0|+) adiabatic charge transition level of $N_{s}$ is 1.7 eV below the conduction band minimum  [30]. Therefore, an electron hop from ${\mathrm{SiV}}^{2-}$ to $N_{s}$ would require an additional ∼1.3 eV (given by 5.47−1.7−2.45 eV), which cannot be supplied by the phonon bath. Even if the auxiliary defects were not substitutional nitrogen but another defect species, the minimal energy mismatch between SiV (2−|−) and the auxiliary defect levels would still be on the order of 1 eV to cover the diamond bandgap. A model supported by density functional theory calculations of the direct non‐local excitation between NV and $N_{s}$ was discussed in Ref.  [31]. Following this model, one can assume that a photon provides the excess of energy required for electron hopping from ${\mathrm{SiV}}^{2-}$ to the $N_{s}^{+}$ (Figure 4a). When this occurs, the remaining ${\mathrm{SiV}}^{-}$ can absorb a photon, promoting an electron from the lying deep in the valence band $a_{2u}$ level to the $e_{g}$ state. The resulting hole on $a_{2u}$ orbital is not localized due to strong mixing with diamond bands  [18]. Usually, this hole relaxes to the $e_{u}$ state, forming the first excited state of the ${\mathrm{SiV}}^{-}$ but under an applied electric field the hole can migrate away from the defect leaving it in the ${\mathrm{SiV}}^{2-}$. We note that this model does not consider the position of the Fermi level with respect to the charge transition levels of defects, but only the deficit of holes that can be retrapped by ${\mathrm{SiV}}^{2-}$. For instance, this can be satisfied if the nearby positive electrode repels holes. Under positive potential, we do not observe luminescence from the SiV center (the *second type*, constituting the majority), which means that the hole drifts away faster than it relaxes to the $e_{u}$ orbital forming ${\mathrm{SiV}}^{2-}$ and no holes recaptured. It also prevents the reverse hopping of the electron from $N_{s}$ to SiV, because all SiV levels are again filled with electrons. $N_{s}$ can be ionized, releasing a free electron and closing the charge conversion cycle. We note that the photocurrent scales linearly with optical power (Figure 4b), although the process involves several photons. It might be counterintuitive unless we assume that multiple auxiliary $N_{s}$ defects are involved (see Supporting Information). The photoionization process starts with some steady state population of $N_{s}$ defects, which is maintained by constant supply of the electrons from the SiV center leading to the linear photoionization behavior. The indication of the involvement of multiple $N_{s}$ rather than a single one that defines the charge stability of NV center was previously shown  [28]. Involvement of multiple $N_{s}$ defects proximal to SiV center does not require a high nitrogen concentration in the sample. We note that the cluster of nearby $N_{s}$ defects together with a SiV defect is much more likely to be introduced during diamond growth than these individual defects because charge transfer from the proximate $N_{s}$ donors toward SiV acceptor defect significantly enhances the probability of incorporation of SiV defect into diamond (see Supporting Information). For instance, the estimated energy gain of a cluster formation of two $N_{s}$ near a SiV defect is about 6.5 eV which can be deduced from previously published data and our new calculations on the charge transition levels of SiV  [18, 19, 30]. These considerations can explain the linear power dependence of the photoionization process which assumes multiple $N_{s}$ defects around SiV.

**FIGURE 4:**
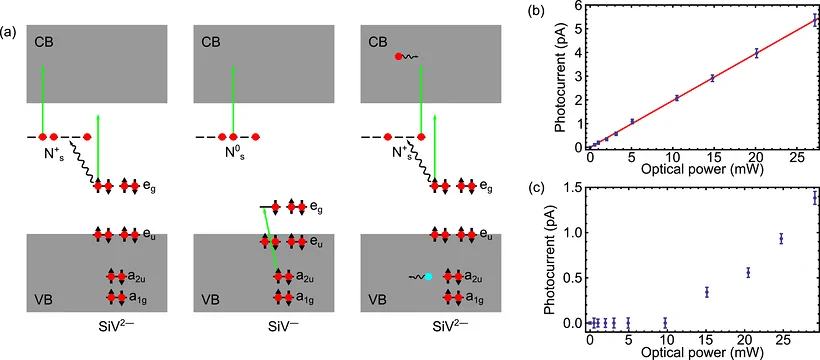
Charge carrier generation. (a) Schematic representation of the charge state conversion cycle, which results in the release of free electrons and holes. Symbol $N_{s}$ denotes levels corresponding to the substitutional nitrogen defects which can accept an electron from the higher orbital of ${\mathrm{SiV}}^{2-}$. The photoionization of these defects can occur at any time, which is indicated as a random ionization act at each stage. (b) Dependence of the photocurrent on excitation OPO power at 455 nm wavelength under positive voltage. (c) Dependence of the photocurrent on excitation OPO power at 455 nm wavelength under negative voltage.

Using first principles calculations, we computed the ${\mathrm{SiV}}^{2-}$+$N_{s}^{+}$ ⟶ ${\mathrm{SiV}}^{-}$+$N_{s}^{0}$ process of which excitation energy depends on the distance between the SiV and $N_{s}$ defects (see Supporting Information). We find that the ZPL energy of this absorption process is ∼1.8 eV at 1.1 nm distance which decreases with the inverse of distance between the defects where the ZPL value approaches ∼1.15 eV at infinitely large distance. We note that the absorption cross section at the ZPL wavelength is minimal because of the giant relaxation energy between $N_{s}^{0}$ with strong Jahn–Teller distorted geometry and $N_{s}^{+}$ with tetrahedral symmetry  [32, 33]. The absorption process is mediated by acoustic phonons where the absorption could be observable by about 0.5 eV above the ZPL energy and it reaches the maximum value at 1.3 eV above the ZPL energy (see Supporting Information for theoretical analysis). By combining the experimental data in Figure 3f with threshold energy at ∼2.3 eV and the first principles dataset for the absorption process, we estimate the distance between the defects at around 1.8 nm where photoexcitation of the donor‐acceptor pair defect at this distance is associated with the rise of the phonon‐assisted absorption band at 2.3 eV (see Figure S11). This estimate is fully consistent with the experimental findings and the expectations from the model.

Under negative potential, the process should be similar. However, the negative potential of the electrode attracts holes from the whole intraelectrode space that facilitates ${\mathrm{SiV}}^{2-}$ in the vicinity of this electrode to capture holes and change the charge state to ${\mathrm{SiV}}^{-}$. After optical excitation of ${\mathrm{SiV}}^{-}$, the hole from the $a_{2u}$ state preferentially relaxes to the $e_{u}$ orbital. Thus, the excited state of ${\mathrm{SiV}}^{-}$ can radiatively decay. Even if the hole is lost in some optical cycle, it can be substituted by another one. During the time intervals, in which SiV is in its negative charge state, reverse electron hopping from $N_{s}$ to ${\mathrm{SiV}}^{-}$ can occurs, converting SiV into ${\mathrm{SiV}}^{2-}$. This process can be interpreted as local recombination of the electrons and holes within the system of SiV and substitutional nitrogen atoms and it competes with the electron‐hole pairs generation process described above. The competing recombination process leads to the net 0 current generated at the SiV position, or might even lead to the local reduction of the background photocurrent (see Supporting Information). With an increase of the optical power, the electron‐hole pair generation process prevails over recombination leading to a weak photocurrent (Figure 4c). The described process explains strong luminescence of ${\mathrm{SiV}}^{-}$ and low photocurrent under negative potential. The behavior of the small subset of SiV centers belonging to the *first type*, for which luminescence and photocurrent are observed simultaneously, is less clear. Thus, the proposed SiV – $N_{s}$ mechanism consistently explains the dominant behavior of the *second‐type* defects, while the simultaneous luminescence and photocurrent observed for the smaller subset of *first‐type* defects likely reflects additional local environmental complexity.

## 4 Conclusion

In summary, we unraveled the complex charge dynamics of SiV centers in diamond showing that the defect can be photoionized and recharged in a closed loop under constant laser illumination, although the photon energy is insufficient to directly promote an electron from SiV to the conduction band. We used the advantage of photoelectric imaging technique to prove that charge conversion and generation of charge carriers occur locally, which means that the previously proposed models suggesting a charge retrap between SiV and auxiliary defect via valence band is incomplete. The observed photoionization cutoff of ${\mathrm{SiV}}^{2-}$ ⟶ ${\mathrm{SiV}}^{-}$ conversion is found to be ∼2.3 eV, which is inconsistent with the theoretically proposed value of 2.95 eV. This suggests the involvement of proximal auxiliary nitrogen donor defects that facilitate photoionization, a hypothesis that is fully supported by our first principles calculations. Moreover, assignment of the defects visible only in photocurrent imaging to the optically inactive ${\mathrm{SiV}}^{2-}$ centers makes it, to the best of our knowledge, the first direct visualization of the point defects which are addressable neither optically nor in electron paramagnetic resonance  [34, 35]. The charge‐conversion process described in our work can be extended to the entire class of group‐IV defects, and this knowledge is important for resolving long‐standing problem: stabilization of the group‐IV defects in the desired charge state. Also of interest is the demonstrated ability to convert, on demand, a small ensemble of color centers into a source of single photons by applying an electric potential, which may relax constrains on creation of defect‐based single photon sources.

## 5 Experimental Section

### 5.1 Experimental Setup

The sample was placed in a home‐built confocal microscope and connected to the bias voltage source (Keithley 487) and a current detection (Femto, gain $10^{11}$) circuit  [36]. The objective from Olympus MPlanApoN 100X, NA 0.95 is used for excitation and collection of fluorescence. This arrangement allowed for simultaneous detection of the confocal and photoelectric raster scans. Raster scans are performed typically using 4 ms integration time per pixel. The photocurrent signal is measured directly, without employing a lock‐in detection scheme. The excitation of the defects was provided by a continuous wave optical parametric oscillator (OPO) from Hübner Photonics CWAVE VIS tunable in a broad range covering 450–525 nm and 540–650 nm. Alternatively, lasers operating at 705 nm (PicoQuant LDH‐D‐C‐705) and home‐built 900 nm were available. The emission collected from the defects could also be analyzed with a spectrometer (Princeton Instruments HRS‐300 with Pixis camera PIX100BX‐SF‐Q‐F‐A). The second‐order autocorrelation functions were measured using the same confocal microscope with the detection system arranged in the Hanbury Brown and Twiss configuration. For optical detection, two avalanche photodiodes from Excelitas SPCM‐AQRH were used. In order to achieve low optical background for antibunching measurements the stack of 725 long pass, 700/100, and 740/10 band pass filters was used. The antibunching measurements were performed with a pulsed laser to avoid a decrease of contrast at zero delay, which is typically observed in continuous‐wave measurements, associated with the short SiV lifetime and jitter of photon‐counting electronics. As an alternative, the detection with the 500 long pass filter was used to probe the fluorescence over a broader spectral range. The characterization of the spectral region around 946 nm was performed on a low‐temperature confocal microscope equipped with a spectrometer (see Supporting Information).

### 5.2 Calculations

We utilized first principles plane‐wave supercell density functional theory (DFT) calculations to study the charge transfer between SiV and $N_{s}$ as implemented in the vasp 5.4.1 code  [37]. We used the usual projector augmented wave (PAW) projectors [38, 39] are applied on the carbon, nitrogen, and silicon atoms with a plane wave cutoff of 370 and 740 eV for charge augmentation cutoff. Our approach requires scaling of the properties as a function of supercell size. In the scaling procedure, supercells of up to 8000 atoms are applied within the semilocal Perdew–Burke–Ernzerhof (PBE) DFT functional  [40] whereas supercells of up to 1728 atoms are employed in the hybrid Heyd–Scuseria–Ernzerhof (HSE) DFT functional  [41, 42]. We optimized the atomic positions individually for every defect geometry until the forces acting on all ions fall behind 0.01 eV/Å while we used a 0.1 meV convergence criteria for each electronic self consistent field (SCF) cycle.

## Author Contributions

The experiments were conducted by I.C. and P.S. with assistance from E.B., M.N. The data were analyzed by I.C. and P.S. with contribution from A.E. and discussed with M.N., A.G., F.J., D.B., A.E. G.T. The DFT calculations were performed by G.T. and A.G. The manuscript was written by P.S., I.C., G.T., A.G. and D.B. with feedback from all authors. P.S., A.G., M.N., F.J. supervised the project.

## Conflicts of Interest

The authors declare no conflicts of interest.

## Supporting information


**Supporting File**: advs76933‐sup‐0001‐SuppMat.pdf.

## Data Availability

The data that support the findings of this study are available from the corresponding author upon reasonable request.

## References

[1] C. Hepp , T. Müller , V. Waselowski , et al., “Electronic Structure of the Silicon Vacancy Color Center in Diamond,” Physical Review Letters 112, no. 3 (2014): 036405, 10.1103/PhysRevLett.112.036405. 24484153

[2] T. Iwasaki , Y. Miyamoto , T. Taniguchi , et al., “Tin‐Vacancy Quantum Emitters in Diamond,” Physical Review Letters 119, no. 25 (2017): 253601, 10.1103/PhysRevLett.119.253601. 29303349

[3] L. J. Rogers , K. D. Jahnke , T. Teraji , et al., “Multiple Intrinsically Identical Single‐Photon Emitters in the Solid State,” Nature Communications 5 (2014): 4739, 10.1038/ncomms5739. 25162729

[4] P. Siyushev , M. H. Metsch , A. Ijaz , et al., “Optical and Microwave Control of Germanium‐Vacancy Center Spins in Diamond,” Physical Review B 96, no. 8 (2017): 081201, 10.1103/PhysRevB.96.081201.

[5] S. Lagomarsino , A. M. Flatae , H. Kambalathmana , et al., “Creation of Silicon‐Vacancy Color Centers in Diamond by Ion Implantation,” Frontiers in Physics 8 (2021): 626, 10.3389/fphy.2020.601362.

[6] A. E. Rugar , C. Dory , S. Aghaeimeibodi , et al., “Narrow‐Linewidth Tin‐Vacancy Centers in a Diamond Waveguide,” ACS Photonics 7, no. 9 (2020): 2356–2361, 10.1021/acsphotonics.0c00833.

[7] E. Bourgeois , A. Jarmola , P. Siyushev , et al., “Photoelectric Detection of Electron Spin Resonance of Nitrogen‐Vacancy Centres in Diamond,” Nature Communications 6 (2015): 8577, 10.1038/ncomms9577. PMC4639813 26486014

[8] M. Niethammer , M. Widmann , T. Rendler , et al., “Coherent Electrical Readout of Defect Spins in Silicon Carbide by Photo‐Ionization at Ambient Conditions,” Nature Communications 10 (2019): 5569, 10.1038/s41467-019-13545-z. PMC6895084 31804489

[9] H. Morishita , S. Kobayashi , M. Fujiwara , et al., “Room Temperature Electrically Detected Nuclear Spin Coherence of NV Centres in Diamond,” Scientific Reports 10 (2020): 792, 10.1038/s41598-020-57569-8. PMC6972904 31964965

[10] M. Gulka , D. Wirtitsch , V. Ivády , et al., “Room‐Temperature Control and Electrical Readout of Individual Nitrogen‐Vacancy Nuclear Spins,” Nature Communications 12 (2021): 4421, 10.1038/s41467-021-24494-x. PMC8292375 34285223

[11] U. F. S. D'Haenens‐Johansson , A. M. Edmonds , B. L. Green , et al., “Optical Properties of the Neutral Silicon Split‐Vacancy Center in Diamond,” Physical Review B 84, no. 24 (2011): 245208, 10.1103/PhysRevB.84.245208. 28949565

[12] S. Dhomkar , P. R. Zangara , J. Henshaw , and C. A. Meriles , “On‐Demand Generation of Neutral and Negatively Charged Silicon‐Vacancy Centers in Diamond,” Physical Review Letters 120, no. 11 (2018): 117401, 10.1103/PhysRevLett.120.117401. 29601766

[13] A. Wood , A. Lozovoi , Z.‐H. Zhang , et al., “Room‐Temperature Photochromism of Silicon Vacancy Centers in CVD Diamond,” Nano Letters 23, no. 3 (2023): 1017, 10.1021/acs.nanolett.2c04514. 36668997

[14] B. L. Green , S. Mottishaw , B. G. Breeze , et al., “Neutral Silicon‐Vacancy Center in Diamond: Spin Polarization and Lifetimes,” Physical Review Letters 119, no. 9 (2017): 096402, 10.1103/PhysRevLett.119.096402. 28949565

[15] B. C. Rose , D. Huang , Z.‐H. Zhang , et al., “Observation of an Environmentally Insensitive Solid‐State Spin Defect in Diamond,” Science 361, no. 6397 (2018): 60–63, 10.1126/science.aao0290. 29976820

[16] M. V. Hauf , B. Grotz , B. Naydenov , et al., “Chemical Control of the Charge State of Nitrogen‐Vacancy Centers in Diamond,” Physical Review B 83, no. 8 (2011): 081304, 10.1103/PhysRevB.83.081304.

[17] B. Grotz , M. Hauf, M. V. Dankerl, B. Naydenov , et al., “Charge State Manipulation of Qubits in Diamond,” Nature Communications 3 (2011): 729, 10.1038/ncomms1729. PMC3316888 22395620

[18] A. Gali and J. R. Maze , “Ab Initio Study of the Split Silicon‐Vacancy Defect in Diamond: Electronic Structure and Related Properties,” Physical Review B 88, no. 23 (2013): 235205, 10.1103/PhysRevB.88.235205.

[19] Z.‐H. Zhang , P. Stevenson , G. Thiering , et al., “Optically Detected Magnetic Resonance in Neutral Silicon Vacancy Centers in Diamond via Bound Exciton States,” Physical Review Letters 125, no. 23 (2020): 237402, 10.1103/PhysRevLett.125.237402. 33337180

[20] C. Schreyvogel , V. Polyakov , R. Wunderlich , J. Meijer , and C. E. Nebel , “Active Charge State Control of Single NV Centres in Diamond by In‐Plane Al‐Schottky Junctions,” Scientific Reports 5 (2015): 12160, 10.1038/srep12160. PMC4503995 26177799

[21] A. T. Collins , “The Fermi Level in Diamond,” Journal of Physics: Condensed Matter 14, no. 14 (2002): 3743–3750, 10.1088/0953-8984/14/14/307.

[22] G. Thiering and A. Gali , “Ab Initio Magneto‐Optical Spectrum of Group‐IV Vacancy Color Centers in Diamond,” Physical Review X 8, no. 2 (2018): 021063, 10.1103/PhysRevX.8.021063.

[23] J. Görlitz , D. Herrmann , P. Fuchs , et al., “Coherence of a Charge Stabilised Tin‐Vacancy Spin in Diamond,” npj Quantum Information 8 (2022): 45, 10.1038/s41534-022-00552-0.

[24] M. Rieger , V. Villafañe , L. Todenhagen , et al., “Fast Optoelectronic Charge State Conversion of Silicon Vacancies in Diamond,” Science Advances 23, no. 8 (2024): eadl4265, 10.1126/sciadv.adl4265. PMC10881026 38381816

[25] J. Walker , “Optical Absorption and Luminescence in Diamond,” Reports on Progress in Physics 42, no. 10 (1979): 1605, 10.1088/0034-4885/42/10/001.

[26] K. Iakoubovskii and G. J. Adriaenssens , “Optical Transitions at the Substitutional Nitrogen Centre in Diamond,” Journal of Physics: Condensed Matter 12, no. 6 (2000): L77–L81, 10.1088/0953-8984/12/6/102.

[27] J. Rosa , J. Pangrác , M. Vanĕc̆ek , et al., “Simultaneous Characterization of Defect States in CVD Diamond by PDS, EPR, Raman and Photocurrent Spectroscopies,” Diamond and Related Materials 7, no. 7 (1998): 1048–1053, 10.1016/S0925-9635(98)00155-1.

[28] N. B. Manson and J. P. Harrison , “Photo‐Ionization of the Nitrogen‐Vacancy Center in Diamond,” Diamond and Related Materials 14, no. 10 (2005): 1705–1710, 10.1016/j.diamond.2005.06.027.

[29] N. B. Manson , M. Hedges , M. S. J. Barson , et al., “ ${\mathrm{NV}}^{-}$–$N^{+}$ Pair Centre in 1b Diamond,” New Journal of Physics 20 (2018): 113037, 10.1088/1367-2630/aaec58.

[30] P. Deák , B. Aradi , M. Kaviani , T. Frauenheim , and A. Gali , “Formation of NV Centers in Diamond: A Theoretical Study Based on Calculated Transitions and Migration of Nitrogen and Vacancy Related Defects,” Physical Review B 89, no. 7 (2014): 075203, 10.1103/PhysRevB.89.075203.

[31] R. Löfgren , S. Öberg , and J. A. Larsson , “A Theoretical Study of De‐Charging Excitations of the NV‐Center in Diamond Involving a Nitrogen Donor,” New Journal of Physics 22 (2020): 123042, 10.1088/1367-2630/abd1ae.

[32] A. Mainwood and A. M. Stoneham , “A Comparison of the Core Exciton and Nitrogen Donor in Diamond,” Journal of Physics Condensed Matter 6, no. 26 (1994): 4917–4927, 10.1088/0953-8984/6/26/013.

[33] E. Londero , E. Bourgeois , M. Nesladek , and A. Gali , “Identification of Nickel‐Vacancy Defects by Combining Experimental and Ab Initio Simulated Photocurrent Spectra,” Physical Review B 97, no. 24 (2018): 241202, 10.1103/PhysRevB.97.241202.

[34] B. G. Breeze , C. J. Meara , X. X. Wu , et al., “Doubly Charged Silicon Vacancy Center, Si‐N Complexes, and Photochromism in N and Si Codoped Diamond,” Physical Review B 101, no. 18 (2020): 184115, 10.1103/PhysRevB.101.184115.

[35] A. Lozovoi , G. Vizkelethy , E. Bielejec , and C. A. Meriles , “Imaging Dark Charge Emitters in Diamond via Carrier‐to‐Photon Conversion,” Science Advances 8, no. 1 (2022): eabl9402, 10.1126/sciadv.abl9402. PMC8741179 34995119

[36] P. Siyushev , M. Nesladek , E. Bourgeois , et al., “Photoelectrical Imaging and Coherent Spin‐State Readout of Single Nitrogen‐Vacancy Centers in Diamond,” Science 363, no. 6428 (2019): 728–731, 10.1126/science.aav2789. 30765564

[37] G. Kresse and J. Furthmüller , “Efficient Iterative Schemes for Ab Initio Total‐Energy Calculations Using a Plane‐Wave Basis Set,” Physical Review B 54, no. 16 (1996): 11169–11186, 10.1103/PhysRevB.54.11169. 9984901

[38] P. E. Blöchl , “Projector Augmented‐Wave Method,” Physical Review B 50, no. 24 (1994): 17953–17979, 10.1103/PhysRevB.50.17953. 9976227

[39] O. Bengone , M. Alouani , P. Blöchl , and J. Hugel , “Implementation of the Projector Augmented‐Wave LDA+U Method: Application to the Electronic Structure of NiO,” Physical Review B 62, no. 24 (2000): 16392–16401, 10.1103/PhysRevB.62.16392.

[40] J. P. Perdew , K. Burke , and M. Ernzerhof , “Generalized Gradient Approximation Made Simple,” Physical Review Letters 77, no. 18 (1996): 3865–3868, 10.1103/PhysRevLett.77.3865. 10062328

[41] J. Heyd , G. E. Scuseria , and M. Ernzerhof , “Hybrid Functionals Based on a Screened Coulomb Potential,” The Journal of Chemical Physics 118, no. 18 (2003): 8207–8215, 10.1063/1.1564060.

[42] A. V. Krukau , O. A. Vydrov , A. F. Izmaylov , and G. E. Scuseria , “Influence of the Exchange Screening Parameter on the Performance of Screened Hybrid Functionals,” Journal of Chemical Physics 125, no. 22 (2006): 224106, 10.1063/1.2404663. 17176133

