# Supplementary material for: Photoelectric Imaging of the Optically Inactive Charge State of Silicon‐Vacancy Defects in Diamond
Source: Adv Sci (Weinh). 2026 Aug 6;13(60):e76933. doi: 10.1002/advs.76933 (PMC13445336; doi:10.1002/advs.76933)
Supplement: Supplementary file 1 [file ADVS-13-e76933-s001.pdf]

Supporting Information for Photoelectrical imaging of the optically inactive charge state of the SiV defects in diamond

Ilia Chuprina,[1] Gergő Thiering,[2] Emilie Bourgeois,[3]
Anna Ermakova,[4,5] Dmitry Budker,[6,7,8,9] Adam Gali,[2,10,11]
Milos Nesladek,[4,3,12] Petr Siyushev,[4,3,1,*] and Fedor Jelezko[1,13]

[1] *Institute for Quantum Optics, Ulm University, D-89081 Germany*

[2] *HUN-REN Wigner Research Centre for Physics, P.O. Box 49, H-1525 Budapest, Hungary*

[3] *IMOMEC division, IMEC, Wetenschapspark 1, B-3590 Diepenbeek, Belgium*

[4] *Institute for Materials Research (IMO), Hasselt University, Wetenschapspark 1, B-3590 Diepenbeek, Belgium*

[5] *Royal Belgian Institute for Space Aeronomy (BIRA-IASB), Ringlaan 3, B-1180, Brussels, Belgium*

[6] *Helmholtz-Institut Mainz, 55099 Mainz, Germany*

[7] *Johannes Gutenberg-Universität Mainz, 55128 Mainz, Germany*

[8] *GSI Helmholtzzentrum für Schwerionenforschung GmbH, 64291 Darmstadt, Germany*

[9] *Department of Physics, University of California, Berkeley, 94720-7300 USA*

[10] *Department of Atomic Physics, Institute of Physics, Budapest University of Technology and Economics, Műegyetem rakpart 3., H-1111 Budapest, Hungary*

[11] *MTA–WFK Lendület "Momentum" Semiconductor Nanostructures Research Group, P.O. Box 49, H-1525 Budapest, Hungary*

[12] *Department of Biomedical Technology, Faculty of Biomedical Engineering, Czech Technical University in Prague, Sítná sq. 3105, 272 01 Kladno, Czech Republic*

[13] *Center for Integrated Quantum Science and Technology (IQST), 89081 Ulm, Germany*

METAL-DIAMOND INTERFACE

For the electrode fabrication, the diamond sample is cleaned for 1.5 hours in a hot mixture of nitric, sulfuric, and perchloric acids. The negative photoresist AZ5214 is spin-coated on the diamond surface and then exposed in the laser writer. After the development of the photoresist, the electrodes are produced on the diamond surface by an electron-beam physical vapor deposition process. A 20 nm layer of titanium is deposited on the diamond surface and then protected with an additional 100 nm layer of gold. Then the photoresist is removed with the AZ100 remover and is additionally cleaned in acetone in an ultrasonic bath. Then the sample is glued to the sample holder and the pads on the diamond and sample holder are connected by wire bonding. The geometry of the contacts is shown in Fig. S1 (a) together with the schematic of the experimental setup. In the experiments, we used areas close to one of the electrodes. This electrode, which is close to the interrogation area, is referred to as the one to which a potential is applied. Near the electrode, the depletion region is formed where band bending occurs. The position of the Fermi level is mainly defined by the nitrogen impurities, a deep donor. Therefore, the Fermi level is lifted above the middle of the bandgap. Because all charge transition levels of SiV are below the

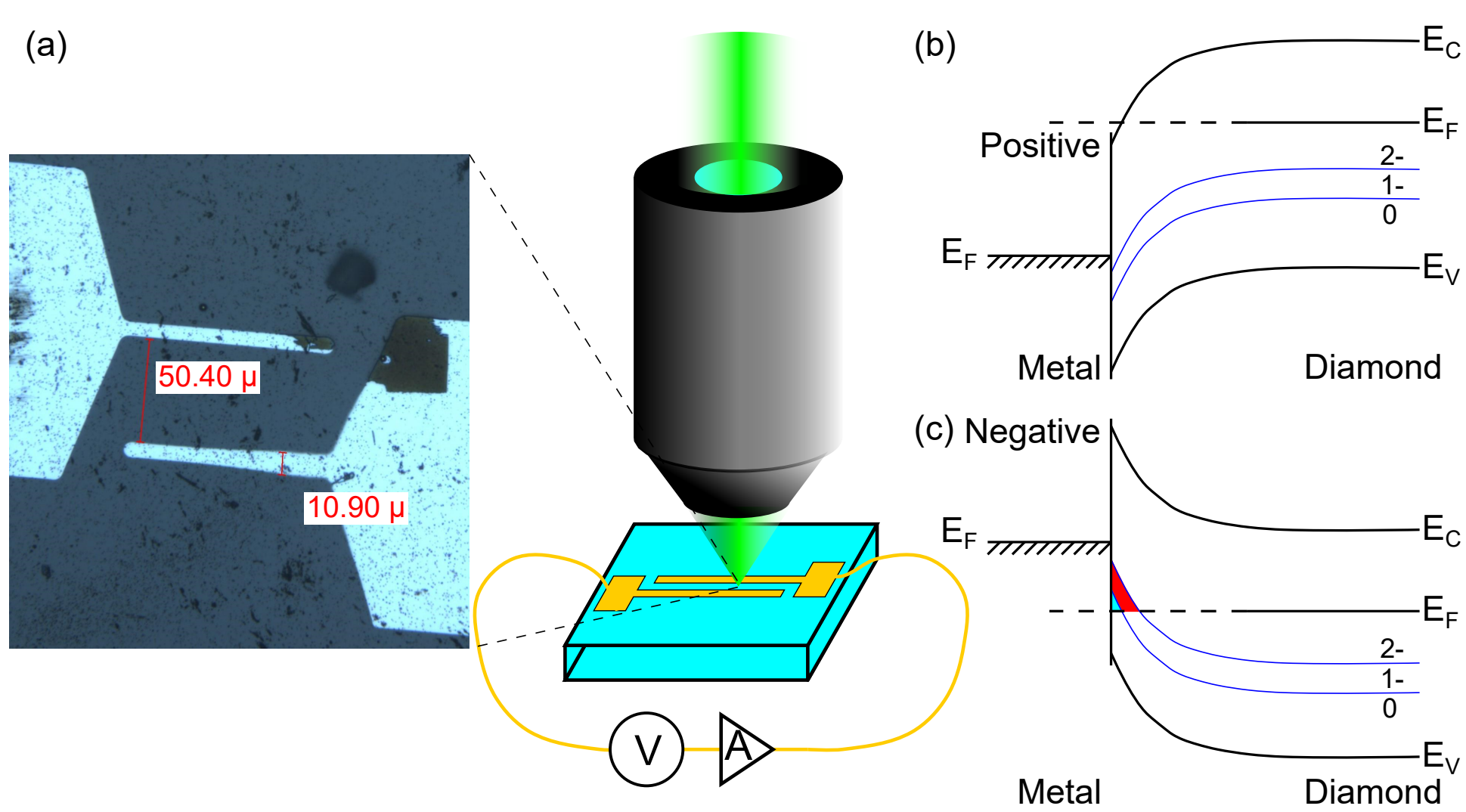

Fig. S1. (a) The geometry of the electrodes and the experimental arrangement. (b) and (c) A schematic band diagram of the titanium-diamond interface under positive and negative potential on the titanium electrode, respectively. Blue lines depict charge transition levels of SiV center. The red (blue) area shows activation of the SiV^- (SiV^0) charge state.

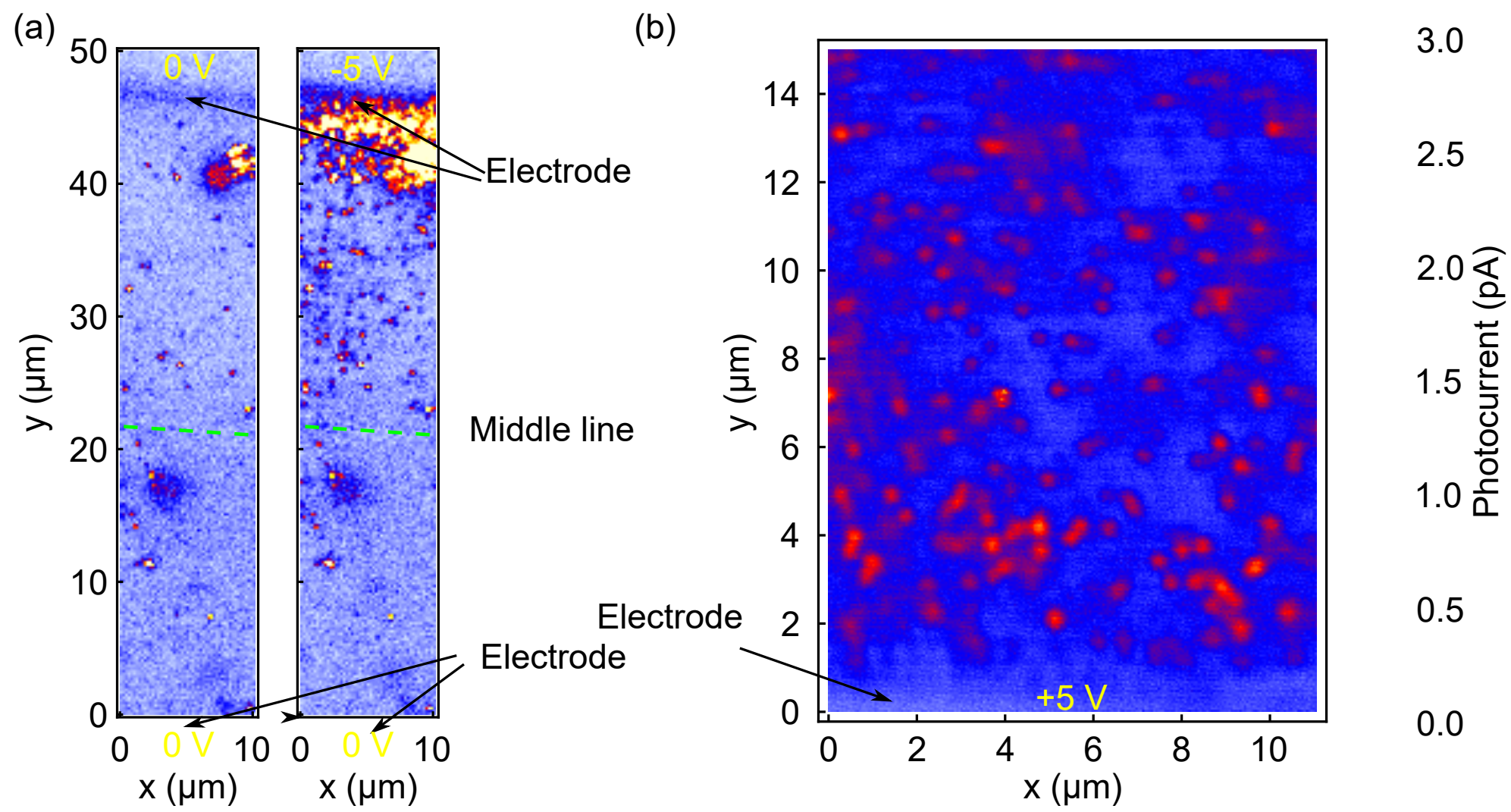

Fig. S2. Example of the switching asymmetry. (a) Photoluminescence maps acquired without bias (left) and with an applied bias of -5 V (right). In the biased case, the luminescence intensity increases near the top electrode and gradually decreases toward the bottom (grounded) electrode. Below the midpoint between the electrodes, none of the SiV centers switches to the luminescent state. There are also large luminescent clusters that are visible in confocal scans for both unbiased and biased cases. However, these clusters do not show photocurrent. (b) Example of a photocurrent-detected map recorded near the positively biased electrode (bottom).

middle of the diamond bandgap, the thermodynamic equilibrium suggests that the SiV has a doubly negative charge. Under positive potential, the downward bending is even increased (Fig. S1 (b)) and thus SiV cannot change its charge state. On the other hand, a negative potential results in upward bending, so that the Fermi level crosses the charge transition levels of SiV center, which can lead to activation of the negative or even neutral charge state (Fig. S1 (c)).

However, such a description typical for semiconductor materials should be carefully considered when applied to such a wide bandgap material as diamond. In diamond, thermal energy cannot activate charge carriers, and thus, the position of the Fermi level without any other changes should not alter the charge states of the defects. Instead of thermal excitation, the laser illumination provides energy to induce charge dynamics. However, band bending only affects the defect charge state in the vicinity of the electrodes within the depletion region. The rough estimation of the depletion region for the nitrogen concentration of $\sim 10^{15}$ cm^{-3}

and the range of voltages in which the experiments were performed does not exceed 5 μm. This matches quite well with the region depicted in Fig. 1 (a) of the main text. However, the switching of the SiV centers also occurs much further away from the electrodes. In our typical experiments, we chose color centers which were at least 10 μm away from the electrode. Therefore, the behavior of the color centers described in this paper cannot be explained by the effect of the band bending alone, so that we attribute it to the excess or deficiency of certain charge carriers near a defect.

The switching is asymmetric with respect to roughly the middle of the interelectrode distance. As seen in Fig. S2 (a), the SiV centers switch to the photoluminescent state in the area from the negatively biased electrode (top) to the middle of the interelectrode space. The switching into the photocurrent state has the same asymmetry with the only difference being that SiV centers switch into this state starting from the positively biased electrode. In Fig. S2 (b), a 15 μm photocurrent map near the positively biased electrode is shown.

SEARCH FOR NEUTRAL SIV CHARGE STATE

In order to understand whether there is a switching of SiV to the neutral charge state under an applied positive voltage, we measured the spectrum centered at around 946 nm. The SiV^0 luminescence is weak and hardly observable at room temperature owing to strong broadening of zero-phonon line [1]. Because the spectra at 4 K under positive potential obtained from the sample used in these studies did not reveal any signature of SiV^0, we utilized a second sample with an ensemble of SiV centers to ensure a strong signal.

The sample was prepared by silicon doping during the plasma-assisted chemical vapor deposition process, resulting in an ensemble of SiV centers. For growth, Element 6 electronic grade diamond was used as a substrate. Both Si and N concentrations during the growth were on the order of 10 ppb.

The titanium-gold electrodes were fabricated in the same manner, and the sample was placed in a cold-finger helium cryostat (CryoVac) combined with a home-built confocal microscope. A 901 nm CW excitation light (C-Wave OPO, Hübner) passing through a 925 nm clean-up filter was focused by the microscope objective lens (Olympus UMPlanFI 100x, NA 0.95) onto the area between the electrodes. The optical power in front of the objective lens was 5.8 mW. The luminescence was filtered with a 937 nm long-pass filter and analyzed with a

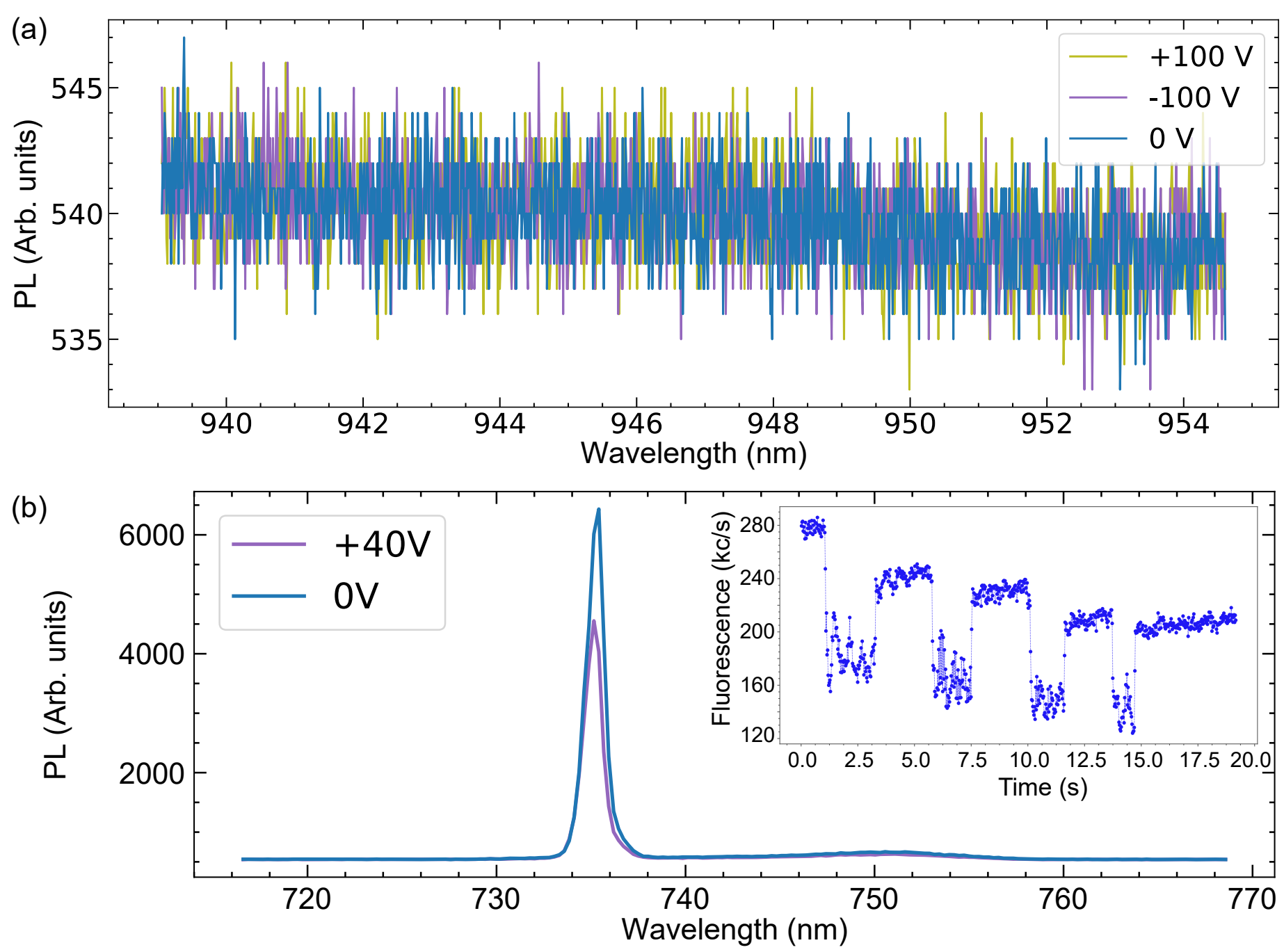

Fig. S3. Spectra of an SiV ensemble. (a) Three representative spectra centered at 946 nm for three different bias voltages: +100, -100 and 0 V. No SiV^0 ZPL (zero-phonon line) at 946 nm is observed within this range of bias voltages. (b) Photoluminescence spectra for the SiV^- detection window taken at two different bias voltages, 0 V (blue) and 40 V (purple), with equal accumulation time. Insert: The optical time trace of SiV^- shows a reduction of counts under an applied positive voltage.

spectrometer (Princeton Instruments with Acton SP2500 0.5 m-long monochromator, grating density of 1596 grooves/mm (BLZ H-NIR), CCD camera PIXIS 100BR with a quantum efficiency of ∼65% at 950 nm). As depicted in Fig. S3(a), no signature of the neutral charge state of SiV was observed in the voltage range of ±100 V. We should note that at the same time, application of voltage changes the photoluminescence of SiV^- centers. In Fig. S3(b), the change of the ZPL (zero-phonon line) amplitude under 0 and +40 V is shown. The inset displays the time trace of the photoluminescence of SiV^- when a positive voltage of +40 V is abruptly switched on and off. Application of positive voltage decreases the photoluminescence. An overall drop in the photoluminescence is visible, associated, presumably, with the loss of holes (or electrons) by the defects in the laser focus due to the drift of charges in the electric field. This means that the steady-state number of the SiV^- centers in the ensemble is changing depending on the applied voltage.

BLINKING NEAR SWITCHING POINT

As discussed in the main text, the centers exhibit blinking behavior in the optical and electrical time traces near the switching point. Visual similarity of the time traces suggests that they have the same origin, which can be proven by the cross-correlation of the electrical and optical signals. However, due to the slow response of the transimpedance amplifier, spikes in the electrical time trace are corrupted by stretched falling edges or even by the absence of some spikes. To minimize these effects and estimate the cross-correlation, the signals were preprocessed. For both traces, switching moments were found with the aid of a hidden Markov model (HMM). The examples of traces with the switching times defined by the HMM are shown in Fig. S4(a) and (b). The cross-correlation was calculated between these HMM-found spikes (Fig. S4(c)), which shows a peak at zero shift. The cross correlation is defined in a standard way for discrete functions: $\sum_{n=0}^{N-1} O(n)E(n+m)$, where $O(n)$ ($E(n)$) is the value of the optical (electrical) signal at a time n.

In some cases, the confocal volume may contain more than one SiV defect, leading to simultaneous or sequential switching of multiple defects. Such behavior was observed as two distinct fluorescence and photocurrent levels during blinking, as shown in Fig. S5. The background and SiV fluorescence levels are indicated by horizontal gray dashed lines. We

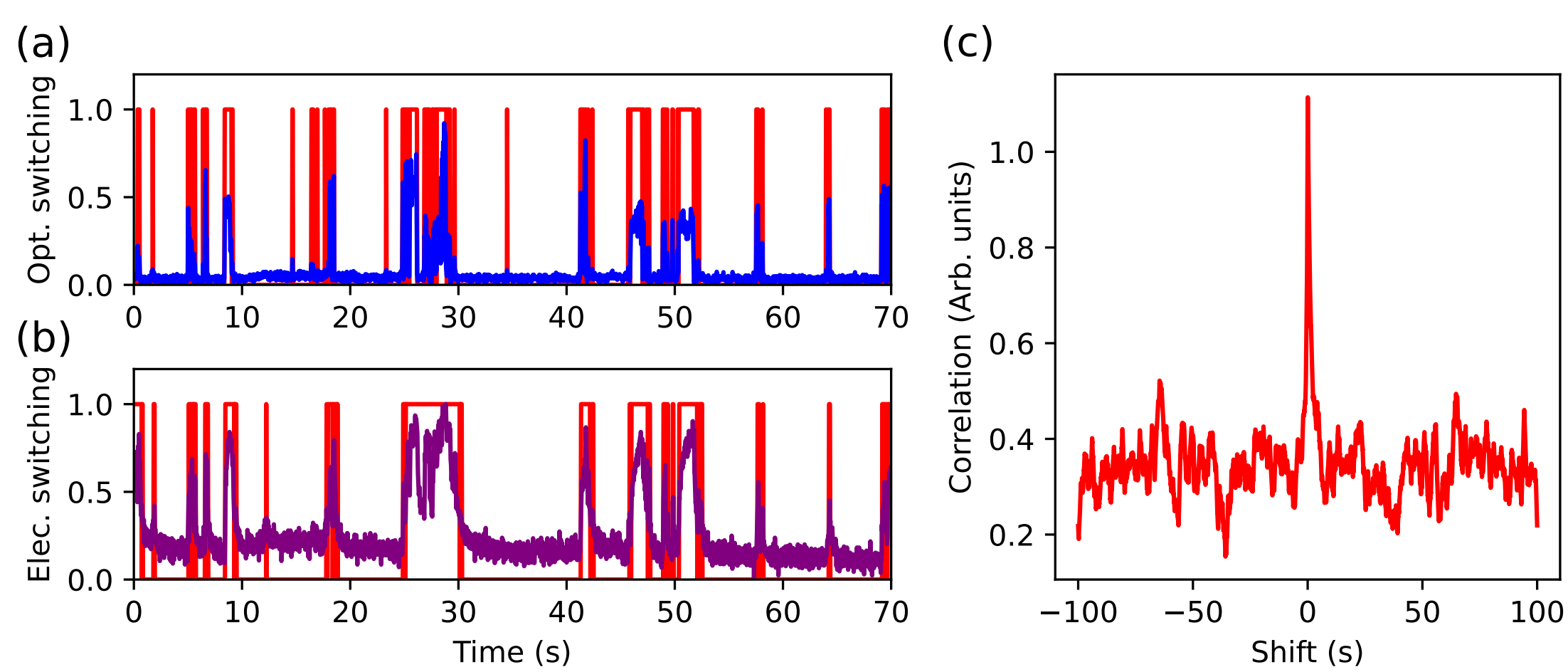

Fig. S4. Cross-correlation of optical and electrical time traces. Examples of (a) optical and (b) electrical time traces with the HMM estimated state switching (red lines). (c) Cross-correlation of the optical and electrical signals.

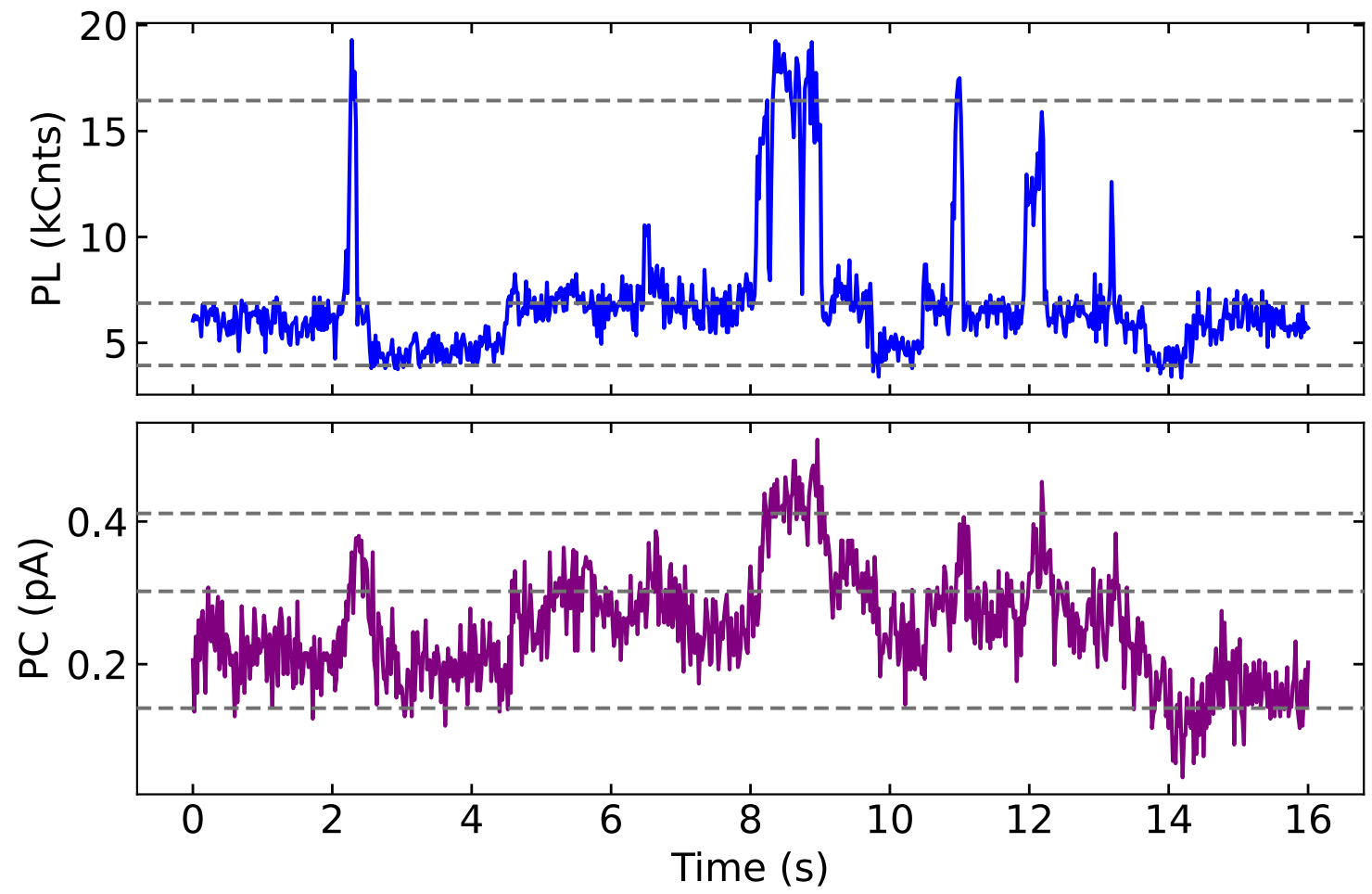

Fig. S5. Blinking in optical (PL) and electrical (PC) signals reveals two distinct signal levels in corresponding optical and electrical traces.

note that, in some cases, a defect may switch only for a short time. Due to the finite response time of the photocurrent signal and fluorescence, not all peaks reach the maximum level during blinking.

PHOTOCURRENT WAVELENGTH THRESHOLD

The wavelength threshold at which the photocurrent vanishes is consistent across multiple defects of the *second type*. For a quantitative analysis, we performed photoelectrical imaging scans containing several isolated defects in a region, which is more than 10 μm away from the electrode. The scans were acquired at a fixed laser excitation power of $\sim$10 mW for each excitation wavelength. From these scans, we analyzed 14 defects that appeared only in photocurrent under a positive bias of +5 V.

An example of a photoelectrical scan is shown in Fig. S6 (a) for excitation wavelengths of 460 nm (left) and 520 nm (right). The photocurrent color scale is kept fixed for both scans, showing a gradual reduction of the signal from spots and the background at longer excitation wavelengths. From the confocal area shown in Fig. S6 (a), we fitted each isolated spot with a Gaussian function and extracted the amplitude and background offset. The photocurrent amplitude extracted for each SiV spot is shown in Fig. S6 (b). As shown in Fig. S6 (b), the

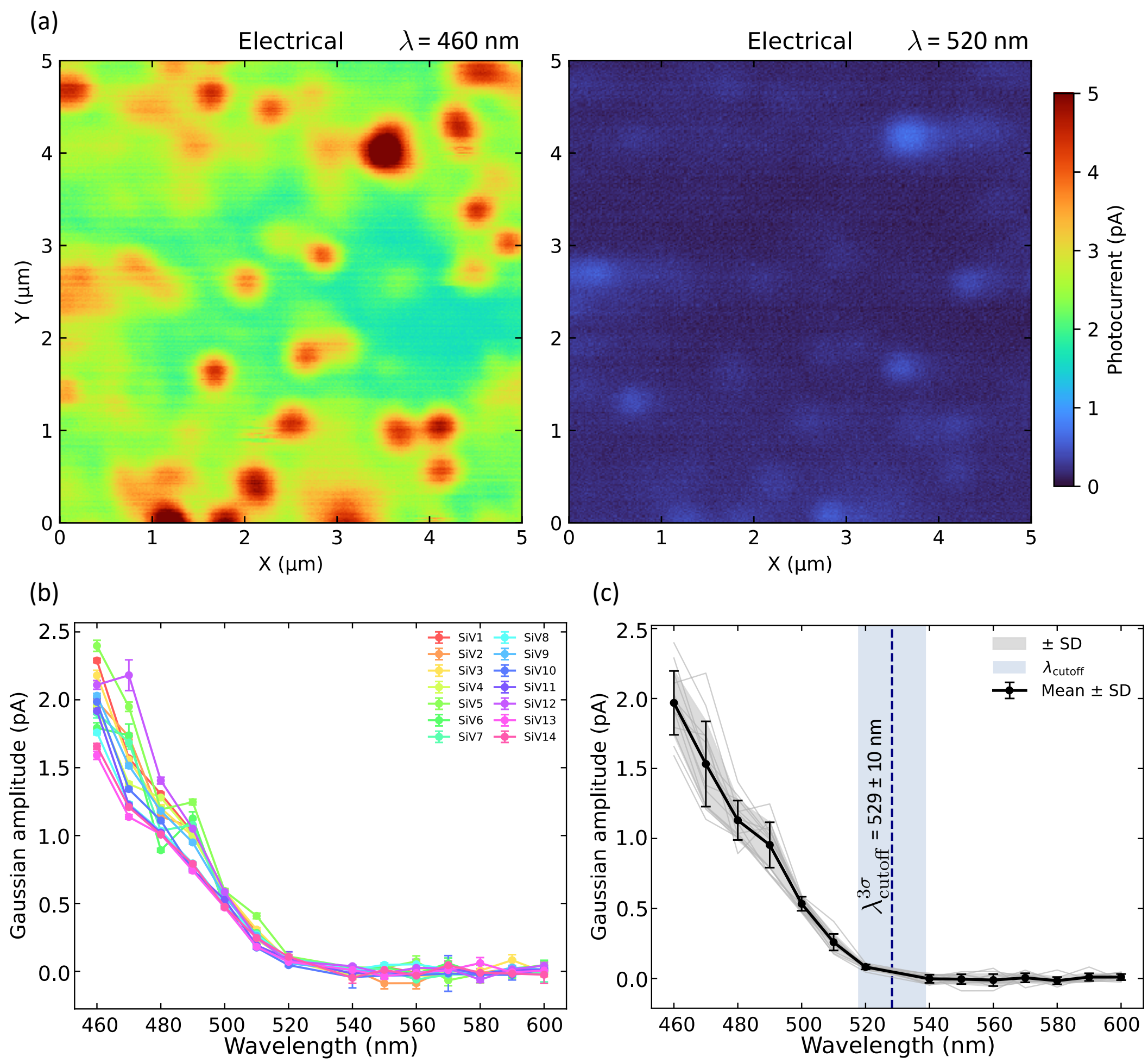

Fig. S6. (a) Photoelectrical confocal maps of the same area acquired at excitation wavelengths of 460 nm (left) and 520 nm (right), both at a laser power of ∼10 mW. The color-bar scale is kept fixed for both confocal scans. (b) Gaussian-fit amplitudes extracted from 14 isolated SiV spots in the corresponding photoelectrical confocal scans. (c) Gaussian-fit amplitudes extracted from the fit for each spot (gray), together with the mean value (black) and standard deviation (black errorbar) over all defects. The 3σ cutoff is depicted as the dark blue dashed line with the light blue shading corresponding to the spot-to-spot standard deviation of the cutoff estimation over 14 SiVs.

amplitudes for all SiVs exhibit a similar gradual decrease and cutoff of the photocurrent with increasing excitation wavelength. In addition, we calculated the mean and standard deviation of the fitted signal across all defects for each excitation wavelength, as shown in Fig. S6 (c). The gray data represent the photocurrent values of individual defects, while the black data points and error bars indicate the corresponding mean and standard deviation.

The data points are connected by solid lines as guides to the eye.

As discussed in the main text and in Supporting Information Sec. and , our interpretation relies on the presence of multiple substitutional nitrogen donors in the vicinity of each SiV center, with a similar average SiV-nitrogen defects separation. A small defect-to-defect variation in the photocurrent is observed. Since each SiV spot is surrounded by a small ensemble of auxiliary nitrogen defects, the relevant parameter is the average SiV-nitrogen distance rather than a discrete SiV-N_s pair separation. Therefore, a discrete dependence of the threshold or photocurrent amplitude is not expected and supported by our measurements.

To provide statistical estimates of the wavelength threshold for a set of SiV defects in Fig. S6 (b), we define the threshold detection in a conventional way as 3 times standard deviation (3σ) plus the long-wavelength mean baseline noise, $I_{bg} + 3\sigma$. The cutoff wavelength was assigned to the first wavelength at which the fitted Gaussian amplitude fell below this threshold and remained below it at longer wavelengths. The cutoff values extracted from all defects were then averaged, and the uncertainty was estimated by combining the spot-to-spot variation with the finite wavelength step of 10 nm. We obtain a mean cutoff wavelength of 529 nm with a spot-to-spot standard deviation of 10.3 nm. The uncertainty is dominated by the spectral sampling and is approximately ± 5 nm. All analyzed spots show that the photoelectric signal vanishes for excitation wavelengths of 540 nm and above. Since the wavelength was sampled in steps of 10 nm, the uncertainty of the cutoff is dominated by the finite sampling interval. We therefore estimate the cutoff depicted in the main text as $\lambda_{cutoff} = 540 \pm 5$ nm.

INFLUENCE OF THE BIAS SIGN

As discussed in the main text, two classes of the SiV defects can be distinguished based on their photocurrent behavior. The first type exhibits photocurrent under positive and negative applied voltage. For the second type of SiV defects, photocurrent depends on the sign of the potential of the closest electrode. In this section, we discuss only the second-type defects. For positive voltage, the photocurrent scales linearly with the optical power. The power dependence depicted in Fig. S7(a) is obtained from the consecutive photoelectric raster

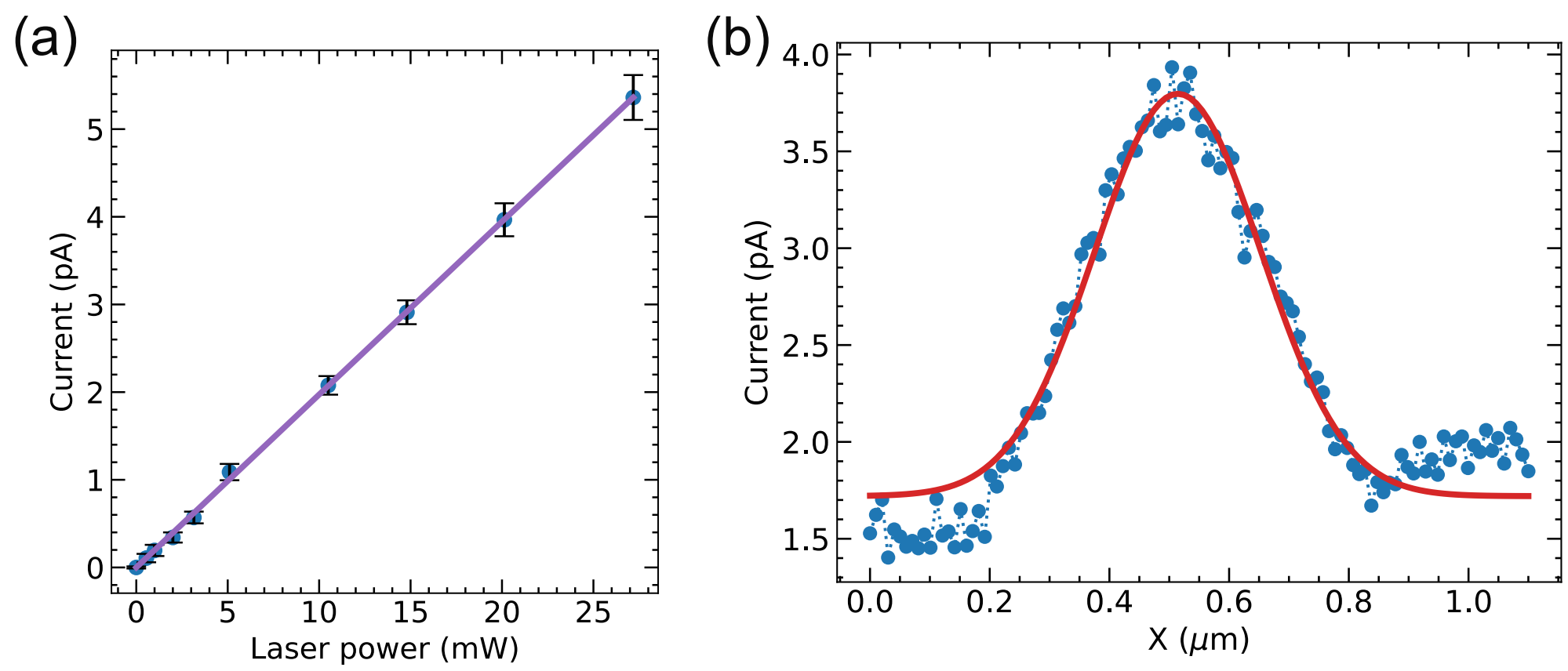

Fig. S7. Photocurrent scaling under positive bias (+5 V, excitation wavelength 455 nm). (a) Linear scaling of the photocurrent with optical power. (b) Cross section through the electrical image of SiV at 10.5mW fitted with a Gaussian function.

scans over the same defect at different laser powers. The cross section through the image of the defect is fitted and the amplitude of the electrical signal from the defect is extracted (Fig. S7(b)). Under negative potential, most of the spots in the photoelectrical image disappear. However, very weak photocurrent from the defects can still be detected, although for many spots this weak signal is completely buried in the noise. To observe this photocurrent, much higher laser power is required (Fig. S8(a)) compared to the case of positive potential (Fig. S7(a)). Interestingly, at moderately low laser power, the photocurrent amplitude at the defect position has the opposite sign, indicating the decrease of the photocurrent at the defect position with respect to the background photocurrent at the surrounding area (Fig. S8(b) left panel). If laser power is increased, the image of the spot changes. When the Gaussian wing of the laser focus starts to overlap with the diffraction-defined area of the spot, the same decrease of the photocurrent is observed. But when the overlap increases (effectively higher power is delivered), the signal turns inside out (Fig. S8(b) middle and right panels). The cross-section of the image (Fig. S8(c)) clearly displays two lobes of the signal. The left lobe corresponds to the reduction of the photocurrent at the beginning of the overlap and the right lobe shows a "normal" increase of the photocurrent with respect to the background. As it is seen from the scans (Fig. S8(b)), the spot is asymmetric. The reason is that the amplitude sign depends also on time, so that this "wrong" component of the amplitude disappears on a tens of milliseconds time scale. Figure S8(a) shows the power dependence of both signal components extracted from the electric raster scans. Please note

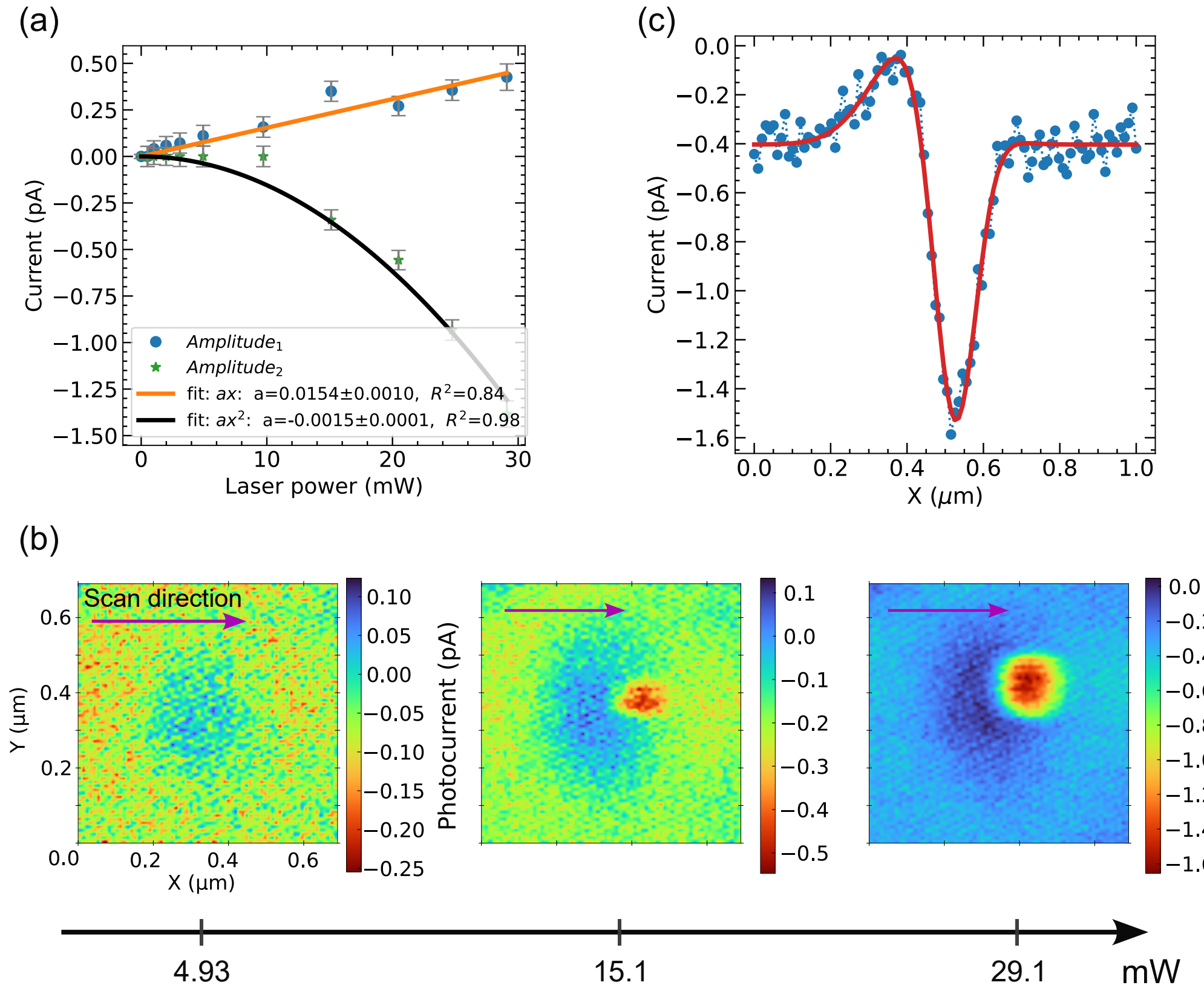

Fig. S8. Photocurrent at a negative bias voltage (-5 V and 455 nm excitation wavelength). (a) Power dependence of the photocurrent amplitudes of positive and negative parts of the signal, extracted from the consecutive raster scans. (b) Examples of raster scans over the same defects at different optical powers. The scan direction is indicated by the arrow. (c) Cross-section of the photoelectric raster image of the defect showing a positive and a negative lobe with respect to the background photocurrent.

that Figure 4(c) of the main text is recorded using a photoelectric time trace where the signal is averaged over 10 s. Therefore, it does not capture the "wrong" part of the signal but rather delayed increase of the photocurrent. The inverted signal with respect to the background indicates that the defects serve as charge traps. Thus, there is a recombination process of the free charge carriers from the background current at the SiV defect. For example, SiV can capture a hole from the valence band while an electron hops from the proximal substitution nitrogen to the SiV, where it effectively recombines. Subsequently, N_S^+ captures an electron from the conduction band and the process can start again. Such process would remove the charge carriers from the total background current flowing through

the diamond. It also might involve other defects which are complexes of Si, N, and vacancies such as SiN, Si_xVN_y [2, 3].

PHOTOCURRENT SCALING

For qualitative understanding of the linear photoionization behavior under an applied positive potential, the fact that the photoluminescence from the SiV centers is completely suppressed should be taken into account. Therefore, conversion into the doubly negative charge state should occur faster than all other processes, particularly, faster than the excited state lifetime of SiV^-, preventing emission of photons. Because SiV is almost all the time is doubly negatively charged, the population of this state $\rho_{SiV^{2-}} \approx 1$. This leads to a very simple model depicted in Figure S9. The electron from the e_g orbital of SiV^{2-} can hop to an N_s using energy from a laser photon. For the population change ρ_{N_s} of N_s, we can write:

$$\dot{\rho}_{N_s} = \eta I \rho_{SiV^{2-}} - \xi I \rho_{N_s}, \tag{S1}$$

where I is the laser intensity, η is the coefficient defining the average hopping rate, and ξ is the coefficient that depends on the photoionization absorption cross section of substitution nitrogen. Note that we assume that there are multiple substitutional nitrogen defects in the SiV surroundings, so that they can always accept an electron from the SiV center. The term with the reversed hopping rate from N_s to SiV^- is neglected, because $\rho_{SiV^-} \approx 0$. The number of electrons per unit of time delivered to the conduction band can be written as

$$\dot{\rho}_{CB} = \xi I \rho_{N_s}. \tag{S2}$$

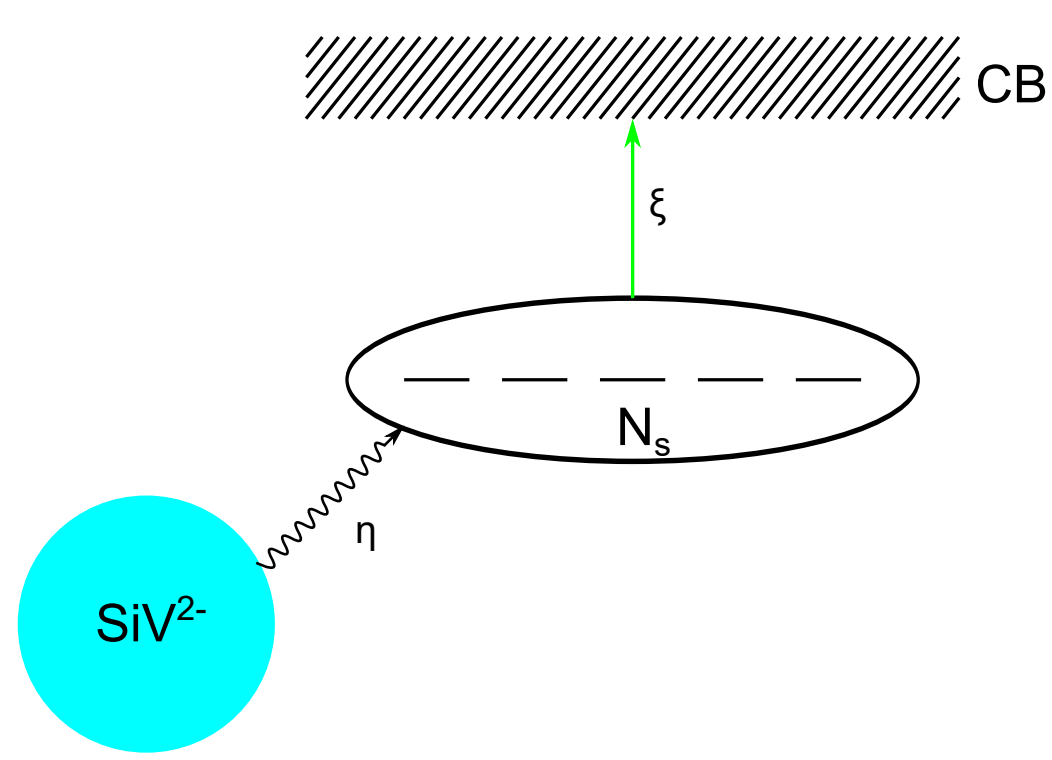

Fig. S9. A simple rate model to explain the linear behavior of photocurrent under positive potential.

We can find ρ_{N_s} from the equation S1, assuming that $\rho_{SiV^{2-}} \approx 1$. The solution in general form is given by

$$\rho_{N_s} = const\, e^{-\xi I t} + \frac{\eta}{\xi}. \tag{S3}$$

The integration constant is defined by the initial population of nitrogen. If it is subjected to light with sufficient energy (above the photoionization threshold), nitrogen is exponentially depleted and the population of N_s is only defined by the ratio of hopping to ionization rates. Substituting equation S3 into S2 we obtain

$$\dot{\rho}_{CB} = const\, \xi I\, e^{-\xi I t} + \eta I. \tag{S4}$$

As noted above, the exponent rapidly decays, leading to the linear dependence of the photocurrent $\sim \eta I$.

SIMULATING THE $SiV^{2-}+N_s^+ \longrightarrow SiV^{-}+N_s^0$ PROCESS

In order to simulate the charge transfer between SiV and N_s, we utilize density functional (DFT) calculations as implemented in the VASP 5.4.1 code [5]. We embed the defect pair into 64-, 216-, 512-, 1000-, 1728-, 2744-, 4096-, 8000-atom diamond supercells [see Fig. S10(b)] within Γ-point sampling of the Brillouin-zone. The standard projector augmented wave (PAW) projectors [6, 7] are applied on N, C, Si atoms with a plane wave cutoff of 370 eV. In the scaling procedure, supercells of up to 8000 atoms are applied within the semilocal Perdew-Burke-Ernzerhof (PBE) DFT functional [4] whereas supercells of up to 1728 atoms are employed in the hybrid Heyd-Scuseria-Ernzerhof (HSE06) DFT functional [8, 9].

We note that the applied supercell model automatically realizes eight immediate N_s neighbors near SiV^{2-} by construction so the $SiV^{2-}+N_s^+$ pair will not be an isolated pair. This will cause a larger overlap between SiV^{2-} and N_s^+ orbitals in this model than that for the truly isolated pair. The numerical error caused by this construction when the total energy of our model is compared to that of the isolated one is in the second order whereas it is in the first order for those quantities which directly depend on the overlap of orbitals. Therefore, we believe that our supercell model well describes the isolated pairs as far as the excitation energies are concerned while the calculated optical transition dipole moment between the ground and excited states is less accurate.

We determine $\mathrm{SiV}^{2-}+\mathrm{N}_\mathrm{s}^+$ pair by non-spin-polarized calculations as both SiV^{2-} and N_s^+ exhibit close-shell singlet states, which is the electronic ground state (gs). We constrain the $\mathrm{SiV}^-+\mathrm{N}_\mathrm{s}^0$ excited state electronic configuration (es) by constraining the net spin as $S = 1$ during a spinpolarized calculation, thus automatically promoting an electron from the occupied SiV related in-gap e_g defect level to the empty $\mathrm{N_s}$ related defect level. We optimized the atomic positions in both cases.

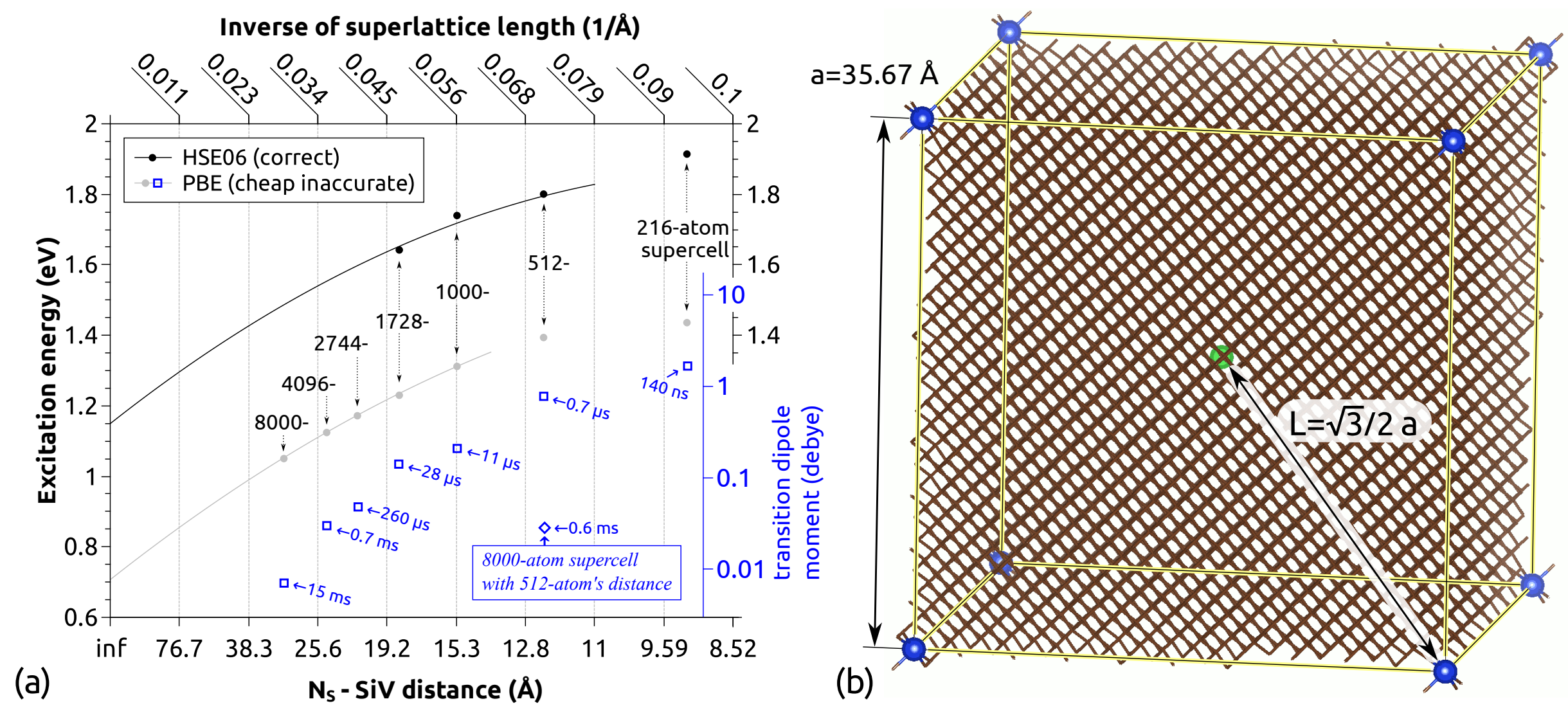

Fig. S10. Non-local excitation of $\mathrm{SiV}^{2-}+\mathrm{N}_\mathrm{s}^+ \to \mathrm{SiV}^-+\mathrm{N}_\mathrm{s}^0$. (a) Firstly, we fit $E_\mathrm{ZPL}\left(\frac{1}{L}\right) = \frac{A}{L^2} + \frac{B}{L} + C$ quadratic function on ab initio results by means of PBE[4] functional to arrive at the $A_\mathrm{PBE} = -14(5)\ \frac{\mathrm{eV}}{\mathrm{Å}^2}$ $B_\mathrm{PBE} = 7.4(8)\ \frac{\mathrm{eV}}{\mathrm{Å}}$ $C_\mathrm{PBE} = 0.72(3)$ eV parameters. We find that parameter B can be identified as the Coulomb repulsion between SiV^{2-} and N_S^+ as follows: $E_\mathrm{Coulomb} = \frac{2e^2}{4\pi\varepsilon_0\varepsilon_r} \times \frac{1}{L} = \frac{7.755\ \mathrm{Å}}{L}$, where $\varepsilon_0 = 0.005526\frac{e}{\mathrm{eVÅ}}$ and $\varepsilon_r = 5.7$ are the dielectric constants of vacuum and the relative permittivity of the diamond host, respectively. We note that PBE functional severely underestimates the optical excitation energies, thus we use the more expensive and more accurate HSE06 functional to simulate the excitation energy within ~ 0.1 eV precision. We again fit a quadratic function but this time we keep $B = 7.755\ \frac{\mathrm{eV}}{\mathrm{Å}}$ as a constant and fit only the $A_\mathrm{HSE06} = -21(4)\ \frac{\mathrm{eV}}{\mathrm{Å}^2}$ and $C_\mathrm{HSE06} = 1.15(5)$ eV parameters. Blue inset depicts the dipole integrals for the $\mathrm{SiV}^{2-}+\mathrm{N}_\mathrm{s}^+$ pair. We calculate the radiative lifetime for the $\mathrm{SiV}^{2-}+\mathrm{N}_\mathrm{s}^+$ pair by means of Eq. (S5). (b) Schematics overview of the 8000-atom supercell hosting 7997 C atoms depicted by brown bonds, one Si atom (blue ball) in the place of two vacancies and one N atom (green ball).

The energy difference between the two electronic configurations, $E_{\mathrm{tot}}^{\mathrm{es}} - E_{\mathrm{tot}}^{\mathrm{gs}}$, goes with the inverse distance $(1/L)$ which is originated from the attractive Coulomb interaction between the positively charged and negatively charged defects in the ground state as depicted in Fig. S10. The extrapolated value for the infinite distance is at 1.15 eV. We note that the photoexcitation from the ground state to the excited state is viable only for close defect pairs because the overlap between the e_g orbital of SiV defect and the empty donor state of $\mathrm{N_s}$ is needed to absorb the photons. Additionally, we calculated the transition dipole moment for the charge transfer within the SiV^{2-}+$\mathrm{N_s^+}$ system. The optical transition can be described by promoting an electron from the fully occupied SiV^{2-} e_g level to the empty $\mathrm{N_s^+}$ a_1 level. Thus, evaluating $\boldsymbol{\mu} = q \times \langle e_{x,y}|\boldsymbol{d}|a_1\rangle$ dipole integrals (Cm in SI units) allows us to approximate the radiative lifetime of a SiV^{-}+$\mathrm{N_s^0}$ pair by utilizing the following formula [10–12]:

$$\tau_{\mathrm{rad}} = \frac{3\pi\varepsilon_0}{n\omega^3|\boldsymbol{\mu}|^2}\,, \tag{S5}$$

where $n = 2.42$ is the refractive index of diamond and we take the $\hbar\omega$ photon energy from extrapolated HSE06 ZPL results; see the black fit curve in Fig. S10(a) for actual data points. We neglect here the Stokes shift for simplicity. We find that the transition dipole moment does not completely vanish even at ∼30 Å of Si-$\mathrm{N_s}$ distances. We find that if the distance is short then the optical lifetime becomes short and the system quickly relaxes to the initial SiV^2- defect near a positively charged nitrogen. Experimentally, activated SiV^--s are stable in the second time scale (see Fig. S4), thus the observed SiV defects do not have too proximate nitrogen neighbors. We note that the defect pairs do not form a superlattice that is generated by the employed method, as depicted in Fig. S10(b). We evaluated the dipole integrals on a 8000-atom supercell with a $\mathrm{SiVN_s}$ distance similarly to the 512-atom case and find that transition probability is overestimated when the wavefunctions overlap from all directions suggesting that the supercell formalism generally overestimates the transition dipole integral in the considered sizes of supercells. Nevertheless, extrapolation for longer distances could lead to useful results. Our model predicts that SiV blinking occurs in the second time scale for such SiV-$\mathrm{N_s}$ pairs that are roughly separated by about $2\ldots4$ nm. At larger distances, the overlap between the relevant wavefunctions of the donor-acceptor pair exponentially decays, which leads to vanishing absorption for SiV-$\mathrm{N_s}$ pairs with a distance of $\gtrsim 5$ nm. As a next step, we study the absorption spectrum.

We find that the optical transition of SiV^{2-}+$\mathrm{N_s^+} \longrightarrow \mathrm{SiV}^{-}$+$\mathrm{N_s^0}$ results in a strong rear-

rangement of ions, in particular, for the N_s part of the pair. The calculated relaxation energy is $E_{\text{Stokes}} = 1.3$ eV. This giant relaxation energy was already recognized for the photoionization of N_s^0 donor [13, 14]. The consequence of the giant relaxation energy upon ionization is that, although, the calculated ZPL energy of photoionization threshold energy is 1.7 eV (e.g., Ref. 15), the observed photoionization occurs at around 2.2 eV (e.g., Refs. 13 and 16) because the absorption probability at ZPL energy is negligible.

This effect can be understood by applying Huang-Rhys theory where the effective single-phonon mode at $\hbar\omega_{\text{phonon}} \approx 65$ meV describes the rearrangement of ions [13] which shifts the on-site nitrogen atom closer to the three neighbor carbon atoms and leaves one carbon dangling bond behind. This Jahn-Teller distorted geometry occurs for N_s^0 and ionization brings the nitrogen atom to on-site position with realizing tetrahedral symmetry. One can estimate the number of effective phonons, i.e., the total Huang-Rhys factor (S), in the photoexcitation by combining E_{Stokes} and $\hbar\omega_{\text{phonon}}$ as $E_{\text{stokes}} = S \times \hbar\omega_{\text{phonon}}$ which yields $S \approx 20$. In this case, the Debye-Waller factor that goes with $\exp(-S)$ is negligible and the phonon assisted absorption band shows a Gaussian lineshape where the maximum of the spectrum is located at the vertical excitation energy, $E_{\text{ZPL}} + E_{\text{Stokes}}$. The absorption spectrum can be generated by the Huang-Rhys theory with calculating the strength of n-th vibration replica, $I_n = \exp(-S)\frac{S^n}{n!}$. At large S factors one can accurately substitute individual I_n phonon overlap integrals with a continuous analytical function as follows,

$$F(\Delta) = \frac{S^{\Delta/\hbar\omega_{\text{phonon}}}}{\Gamma(\Delta/\hbar\omega_{\text{phonon}} + 1)}, \tag{S6}$$

where $\Delta = n \times \hbar\omega_{\text{phonon}}$ is the energy of n phonons. We note that we also replaced the factorial $n!$ with the Gamma function: $\Gamma(n+1)$. Finally, we take into consideration that the absorption of the photon increases linearly with its frequency. Thus, the probability of the charge transfer (i.e., absorption of the photons) is

$$I(\Delta) = \Delta \times F(\Delta - E_{\text{ZPL}}) = \Delta \times \frac{S^{(\Delta - E_{\text{ZPL}})/\omega_{\text{phonon}}}}{\Gamma\left(\frac{\Delta - E_{\text{ZPL}}}{\hbar\omega_{\text{phonon}}} + 1\right)}\,. \tag{S7}$$

which we plot in Fig. S11 as a function of the distance of the nearby defect pairs. We note that Δ in Eq. (S7) now depicts the energy of the absorbed photon that induces the photocurrent. The yellow vertical line depicts the observed photoionization threshold energy in Fig. 3(f) which indicates that the photoionization already occurs for defect pairs with

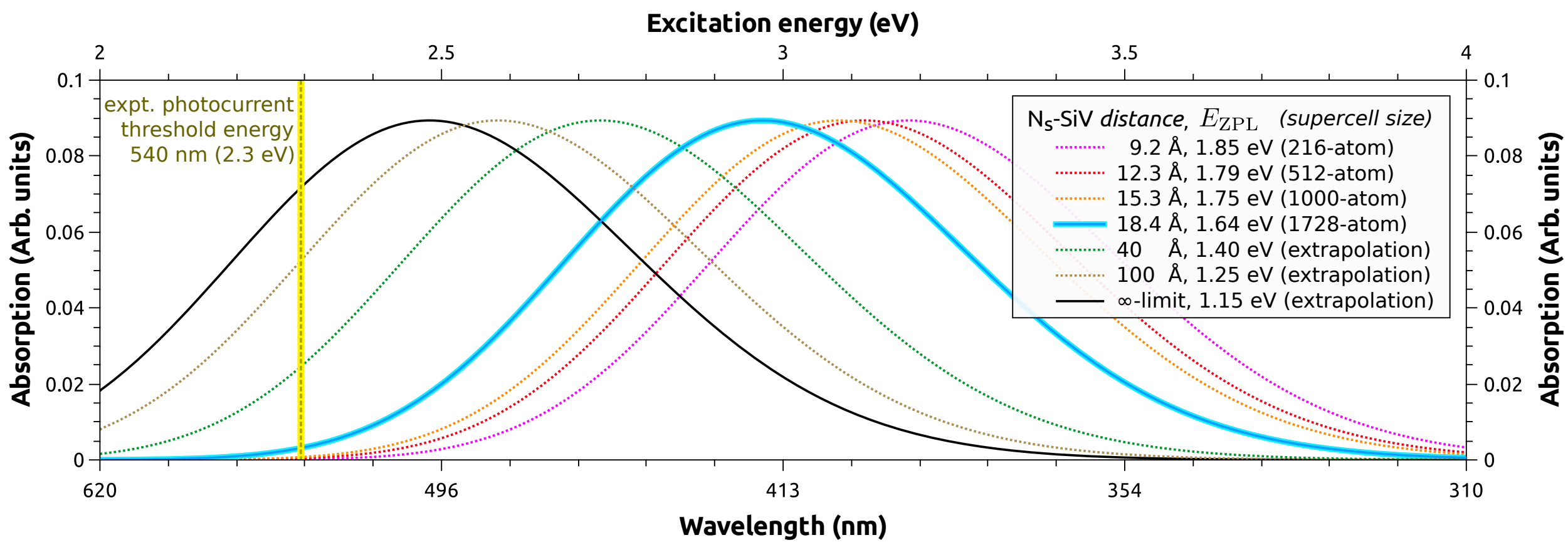

Fig. S11. Probability of $\mathrm{SiV}^{2-}+\mathrm{N}_\mathrm{s}^{+} \longrightarrow \mathrm{SiV}^{-}+\mathrm{N}_\mathrm{s}^{0}$ charge transfer with respect to the wavelength of absorbed photons as a function of the distance between the defect pairs by means of Eq. (S7). We used $E_\mathrm{stokes} = 1.3$ eV and $\hbar\omega_\mathrm{phonon} = 65$ meV, thus $S = 20$ parameters for all plots. We take the HSE06 E_ZPL values from Fig. S10 for 216-, 512-, 1000-, 1728- atom supercells and extrapolations at 40 Å, 100 Å, $+\infty$ $\mathrm{N_s} - \mathrm{SiV}$ distances. The vertical line is the observed photocurrent threshold energy at 540 nm (see the main text). We note that, we show the blue highlighted 1728-atom case in Fig. 3.(f) of the main text for comparison with experimental data. We additionally note the curves are approximately Gaussians with maximums at $E_\mathrm{stokes} + E_\mathrm{ZPL}$ excitation energies.

~ 2 nm distance.

FORMATION OF NEARBY SIV AND NITROGEN DONOR DEFECTS DURING GROWTH

We consider the formation of SiV and nitrogen defects in the CVD growth of diamond. Although, the CVD growth of diamond is not an equilibrium process, the formation energy of defects is still an approximate indicator for their concentration. SiV is a double acceptor [17] whereas $\mathrm{N_s}$ is a single donor (e.g., Ref. 15). As a consequence, one $\mathrm{N_s}$ defect can ionize a SiV defect once, whereas two $\mathrm{N_s}$ defects could double ionize a SiV defect. On the other hand, diamond is a wide band gap material in which the defects exhibit deep ionization energies, and for a low concentration of impurities, the Fermi level does not unambiguously define the defect's charge state [18]. On the other hand, CVD growth of diamond typically

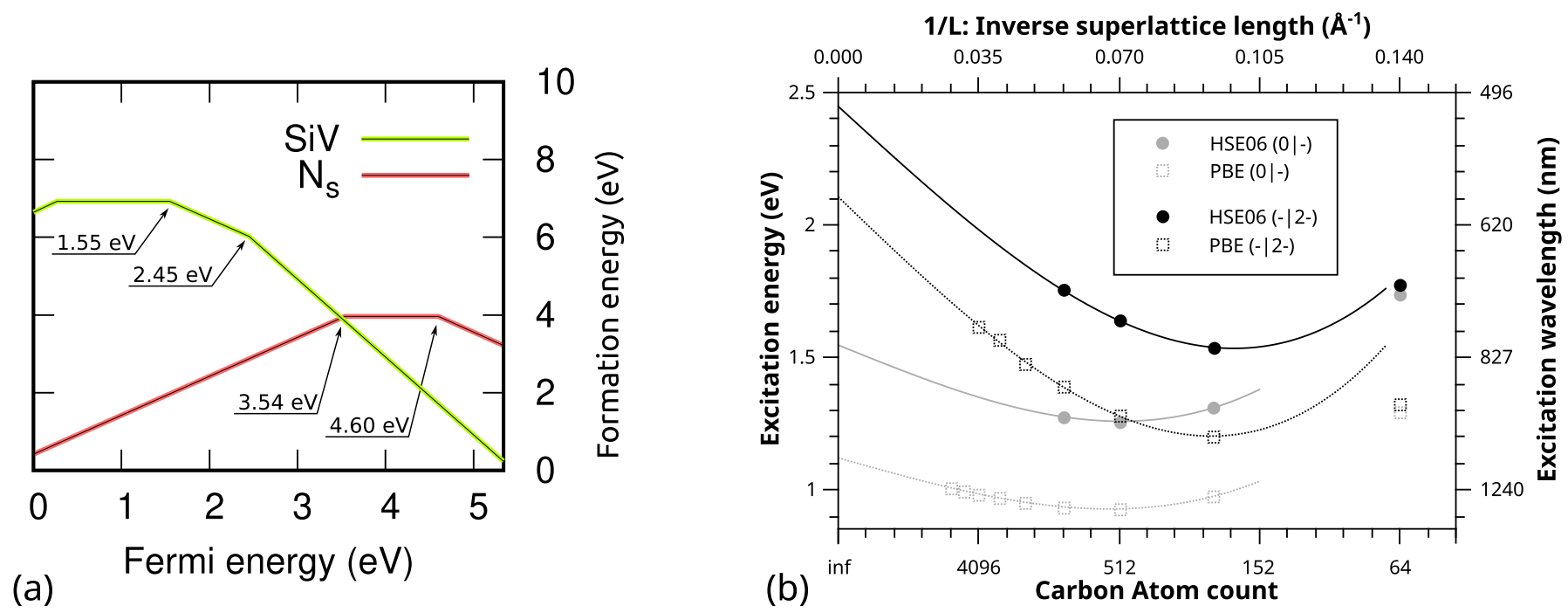

Fig. S12. Formation energies and charge transition levels of SiV and N_s defects in diamond. (a) Formation energy of defects as a function of the Fermi-level respect to the valence band maximum (VBM) using accurate size correction. We take the formation of neutral SiV and N_s from Refs. [19] and [15] respectively. We use the charge transition levels of N_s from Ref. [15]. We take extrapolated ($L^{-1} \to 0$) charge transition levels of SiV from (b) except the (+|0) case that we take from Ref. [19] at VBM+0.265 eV for simplicity. (b) Simulated charge transition levels of SiV(0|−) and SiV(−|2−) by means of density functional calculations hosting only one SiV defect in the superlattice. We fitted quadratic functions on our data points (inverse superlattice length) except the 64-atom case. Therefore, we expect the SiV(0|−) charge transition level at VBM+1.55 eV and SiV(−|2−) at VBM+2.45 eV.

occurs at elevated temperatures that may mediate exchange of electrons between nearby defects in diamond. However, if these defects are far from each other, then all the defects may remain in their neutral charge state. The formation energies of defects are plotted in Fig. S12(a). We note that we have recently recognized that accurate (−|0) charge transition level of SiV in diamond can only be obtained by extrapolation scheme for the charged defective supercells [20] that yields 1.51 eV above the valence band maximum. Therefore, we here reiterated this scheme to the (−2|−) charge transition level of SiV which resulted in 2.45 eV above the valence band maximum as shown in Fig. S12(b). These acceptor levels lie somewhat higher than those reported in a previous study [17]. We plot the formation energy of SiV defect with these improved values and taking the previous *ab initio* data from Ref. 15 for N_s.

The formation energies of remote SiV^0 and N_s^0 are 6.9 eV and 4.0 eV, respectively, so the total cost of introducing two remote defects is 10.9 eV. By considering a nearby SiV^-

and N_s^+ defect pair, the charge transfer of the electron from N_s towards SiV will effectively reduce the formation energies of both species which yields 8.8 eV. As a consequence, the energy gain for introducing this defect pair is about 2.1 eV. By considering nearby SiV^{2-} and two N_s^+, the formation energy of this cluster is 11.6 eV which is 3.3 eV lower than that of remote neutral species (14.9 eV). These energy gains mean orders of magnitude in terms of concentration of defects. In other words, it is much more likely to find a SiV defect near one substitutional nitrogen defect than isolated ones, and it is even more likely to find a SiV defect near two substitutional nitrogen defects than isolated ones or the defect pair provided that Si and N species are available in the CVD growth chamber. This may explain why the incorporation of SiV defect during CVD growth of diamond dominantly could occur together with two nitrogen donors.

The data from DFT calculations that support the findings of this study are directly available from Gergő Thiering (email: thiering.gergo@wigner.hun-ren.hu) upon reasonable request.

---

* petr.siyushev@uhasselt.be

[1] U. F. S. D'Haenens-Johansson, A. M. Edmonds, B. L. Green, M. E. Newton, G. Davies, P. M. Martineau, R. U. A. Khan, and D. J. Twitchen, Optical properties of the neutral silicon split-vacancy center in diamond, Phys. Rev. B **84**, 245208 (2011).

[2] B. G. Breeze, C. J. Meara, X. X. Wu, C. P. Michaels, R. Gupta, P. L. Diggle, M. W. Dale, B. L. Cann, T. Ardon, U. F. S. D'Haenens-Johansson, I. Friel, M. J. Rayson, P. R. Briddon, J. P. Goss, M. E. Newton, and B. L. Green, Doubly charged silicon vacancy center, Si-N complexes, and photochromism in N and Si codoped diamond, Phys. Rev. B **101**, 184115 (2020).

[3] M. R. Zemła, K. Czelej, P. Kamińska, C. G. Van de Walle, and J. A. Majewski, Electronic structure and magneto-optical properties of silicon-nitrogen-vacancy complexes in diamond, Phys. Rev. B **102**, 115102 (2020).

[4] J. P. Perdew, K. Burke, and M. Ernzerhof, Generalized Gradient Approximation Made Simple, Phys. Rev. Lett. **77**, 3865 (1996).

[5] G. Kresse and J. Furthmüller, Efficient iterative schemes for ab initio total-energy calculations

using a plane-wave basis set, Phys. Rev. B **54**, 11169 (1996).

[6] P. E. Blöchl, Projector augmented-wave method, Phys. Rev. B **50**, 17953 (1994).

[7] O. Bengone, M. Alouani, P. Blöchl, and J. Hugel, Implementation of the projector augmented-wave LDA+U method: Application to the electronic structure of NiO, Phys. Rev. B **62**, 16392 (2000).

[8] J. Heyd, G. E. Scuseria, and M. Ernzerhof, Hybrid functionals based on a screened Coulomb potential, J. Chem. Phys. **118**, 8207 (2003).

[9] A. V. Krukau, O. A. Vydrov, A. F. Izmaylov, and G. E. Scuseria, Influence of the exchange screening parameter on the performance of screened hybrid functionals, J. Chem. Phys. **125**, 224106 (2006).

[10] V. Weisskopf and E. Wigner, Berechnung der natürlichen linienbreite auf grund der diracschen lichttheorie, Z. Phys. **63**, 54–73 (1930).

[11] G. Thiering and A. Gali, Ab initio magneto-optical spectrum of group-IV vacancy color centers in diamond, Phys. Rev. X **8**, 021063 (2018).

[12] G. Thiering and A. Gali, Erratum: Ab initio magneto-optical spectrum of group-IV vacancy color centers in diamond [Phys. Rev. X 8, 021063 (2018)], Phys. Rev. X **10**, 039901 (2020).

[13] A. Mainwood and A. M. Stoneham, A comparison of the core exciton and nitrogen donor in diamond, J. Phys. Condens. Matter **6**, 4917–4927 (1994).

[14] E. Londero, E. Bourgeois, M. Nesladek, and A. Gali, Identification of nickel-vacancy defects by combining experimental and ab initio simulated photocurrent spectra, Phys. Rev. B **97**, 241202 (2018).

[15] P. Deák, B. Aradi, M. Kaviani, T. Frauenheim, and A. Gali, Formation of NV centers in diamond: A theoretical study based on calculated transitions and migration of nitrogen and vacancy related defects, Phys. Rev. B **89**, 075203 (2014).

[16] K. Iakoubovskii and G. J. Adriaenssens, Optical transitions at the substitutional nitrogen centre in diamond, J. Phys.: Condens. Matter **12**, L77–L81 (2000).

[17] A. Gali and J. R. Maze, Ab initio study of the split silicon-vacancy defect in diamond: Electronic structure and related properties, Phys. Rev. B **88**, 235205 (2013).

[18] A. T. Collins, The fermi level in diamond, J. Phys.: Condens. Matter **14**, 3743–3750 (2002).

[19] G. Thiering and A. Gali, Complexes of silicon, vacancy, and hydrogen in diamond: A density functional study, Phys. Rev. B **92**, 165203 (2015).

[20] Z.-H. Zhang, P. Stevenson, G. Thiering, B. C. Rose, D. Huang, A. M. Edmonds, M. L. Markham, S. A. Lyon, A. Gali, and N. P. de Leon, Optically detected magnetic resonance in neutral silicon vacancy centers in diamond via bound exciton states, Phys. Rev. Lett. **125**, 237402 (2020).
